# Genetic analysis of capsular polysaccharide synthesis gene clusters in 79 capsular types of *Klebsiella* spp

**DOI:** 10.1038/srep15573

**Published:** 2015-10-23

**Authors:** Yi-Jiun Pan, Tzu-Lung Lin, Chun-Tang Chen, Yi-Yin Chen, Pei-Fang Hsieh, Chun-Ru Hsu, Meng-Chuan Wu, Jin-Town Wang

**Affiliations:** 1 Department of Microbiology, National Taiwan University College of Medicine, Taipei, Taiwan; 2 Department of Internal Medicine, National Taiwan University Hospital, Taipei, Taiwan

## Abstract

A total of 79 capsular types have been reported in *Klebsiella* spp., whereas capsular polysaccharide synthesis (*cps*) regions were available in only 22 types. Due to the limitations of serotyping, complete repertoire of *cps* will be helpful for capsular genotyping. We therefore resolved the rest 57 *cps* and conducted comparative analysis. Clustering results of 1,515 predicted proteins from *cps* loci categorized proteins which share similarity into homology groups (HGs) revealing that 77 Wzy polymerases were classified into 56 HGs, which indicate the high specificity of *wzy* between different types. Accordingly, *wzy*-based capsular genotyping could differentiate capsule types except for those lacking *wzy* (K29 and K50), those sharing identical *wzy* (K22 vs. K37); and should be carefully applied in those exhibited high similarity (K12 vs. K41, K2 vs. K13, K74 vs. K80, K79 vs. KN1 and K30 vs. K69). Comparison of CPS structures in several capsular types that shared similarity in their gene contents implies possible functions of glycosyltransferases. Therefore, our results provide complete set of *cps* in various types of *Klebsiella* spp., which enable the understandings of relationship between genes and CPS structures and are useful for identification of documented or new capsular types.

The genus *Klebsiella*, especially the species *Klebsiella pneumoniae*, is a common human pathogen that can lead to a wide range of diseases in both hospital and community settings. It causes nosocomial infections, such as septicemia, pneumonia, and urinary tract infections^1^,^2^, and is also associated with community-acquired infections, including pneumonia, urinary tract infections and pyogenic liver abscess complicated with meningitis and endophthalmitis^3^,^4^,^5^. Capsule is a major virulence factor of *K. pneumoniae*, and association between capsular types and particular diseases^6^,^7^ or severity of infections was documented^8^,^9^. At present, a total of 79 capsular types have been identified and associated with different *Klebsiella* species^10^, including 77 types from reference strains (recently reclassified into *K. pneumoniae, K. variicola, K. oxytoca, K. michiganensis, Raoultella planticola, R. ornithinolytica*, and *R. terrigena*^10^) recognized by serological reactivity tests established during the period 1926 to 1977^11^ and 2 new types of *K. pneumoniae* (KN1 and KN2) characterized by molecular genotyping and phage typing in recent years^12^,^13^.

Serotyping has been used to characterize the K-types of *Klebsiella* spp. since 1926^14^. However, the limitations of serotyping of *Klebsiella* spp. have been reported in several studies, including limitations of sensitivity and specificity^15^,^16^,^17^. For this reason, capsular genotyping methods have been developed for discriminating the capsular types of *Klebsiella* spp.^10^,^12^,^18^,^19^,^20^,^21^,^22^,^23^,^24^. Polymerase chain reaction-based genotyping of the capsular polysaccharide synthesis region, *cps*-PCR genotyping, was first adopted for the detection of specific *wzy* genes in *Klebsiella* spp. type K1^18^,^19^,^20^ and subsequently applied to other capsular types related to community-acquired pyogenic liver abscess^12^,^21^,^22^. Recently, *wzi* or *wzc* sequencing was also used for *Klebsiella* spp. capsular typing^10^,^25^. However, some types were undistinguished by their sequences and it can be also complicated to determine the capsular types when sequence variation exists in a given type.

Genetic structures of the capsular polysaccharide synthesis (*cps*) gene cluster in *Klebsiella* spp. have been determined in some types^12^,^18^,^26^,^27^,^28^. A group of six genes (*galF, cpsACP, wzi, wza, wzb* and *wzc*) at the 5′ end of the *cps* regions that encode proteins involved in CPS translocation and processing at the bacterial surface are highly conserved among different capsule types and genes encoding glucose-6-phosphate dehydrogenase (*gnd*) and UDP-glucose dehydrogenase (*ugd*) were found at the 3′ end. The middle region (variable region) of the *cps* loci which comprises particular genes encoding proteins responsible for polymerization and assembly of specific CPS subunits were therefore considered to be crucial for K-type variation^18^. Generally, the synthesis of the capsular repeat is initiated by the initial glycosyltransferase (GT)–WbaP and WcaJ^28^,^29^ and further catalyzed by specific (non-initial) GTs allowing the addition of sugars^29^. The lipid-linked repeat units are flipped across the plasma membrane by Wzx and then polymerized by Wzy^30^. Subsequently, the channel Wza, together with regulators Wzb and Wzc, which control the process of polymerization and transportation, exports the polymer to the surface of the bacteria^29^.

Of the 79 documented capsular types in *Klebsiella* spp., the *cps* gene clusters of 22 types (complete 13 *cps* for K1, K2, K3, K5, K9, K14, K16, K20, K22, K39, K52, K62 and KN2; incomplete 9 *cps* for K15, K23, K37, K45, K50, K54, K57, K79 and KN1) are available^6^,^12^,^18^,^25^,^28^. In order to associate all of 79 *cps* gene clusters with distinct capsular types, we sequenced the *cps* of 57 capsular types of *Klebsiella* spp. and extend the 3′ of incomplete 9 *cps*, and conducted comparative analysis of the *cps* gene clusters of various types. Investigation into the relationships between different capsular gene clusters provided further understanding of capsule biosynthesis. Moreover, as we have gained more complete information on the genetic structures of all 79 capsular types, the limitations of current genotyping methods can be more clearly defined and the use of these typing methods can be further improved.

## Results

### *cps* gene clusters of 79 *Klebsiella* spp. capsular types

We obtained all the 79 *cps* gene clusters which extend from *galF* to *ugd* (except for K4 and K50) by retrieving sequences from Genbank database (13 complete *cps* and 9 incomplete *cps*), extending the 3′ *cps* sequences in the 9 types and resolving 57 *cps* of *Klebsiella* spp. (Supplementary Table S1). In K4, we failed to extend the downstream sequences of *gnd*; in K50, conserved *gnd* or *ugd* genes were not found in this locus although a ~21 kb region from *galF* to the downstream genes *HisA* and *HisF* (which encode enzymes associated with histidine biosynthesis and are generally located downstream of *cps* gene clusters in *Klebsiella* spp.^18^) was resolved. Moreover, we identified *K. pneumoniae* strain BIDMC 47 as K13 by *wzc* genotyping^25^ (100% DNA sequence identity). Thus, the full *cps* sequences of BIDMC47 (accession number AB924555) was included to represent K13 type. For these 78 *cps*, a total of 1515 coding sequences were annotated, including *galF* (n = 79), *cpsACP* (n = 80), *wzi* (n = 78), *wza* (n = 81), *wzb* (n = 78), *wzc* (n = 78), *gnd* (n = 79), *manB* (n = 43), *manC* (n = 44), *rmlA* (n = 30), *rmlB* (n = 29), *rmlC* (n = 30), *rmlD* (n = 30), *wcaJ* (n = 40), *wbaP* (n = 39), *gmd* (n = 6), *wcaG* (n = 6), *glf* (n = 5), *wzx* (n = 77), *wzy* (n = 77) and genes encoding non-initial GTs (n = 318), glycosyl hydrolase (n = 33), acetyltransferases (n = 35), pyruvyltransferases (n = 35), transposases (n = 21), nitroreductase (n = 2), potassium/proton antiporter (n = 2), tail fiber (n = 6), acetylneuraminic acid synthetase (n = 1), UDP galacturonate 4-epimerase (n = 1), carbohydrate lyase (n = 1), CMP-N-acetylneuraminic acid synthetase (n = 1), coenzyme F420 hydrogenase (n = 1) and hypothetical proteins (n = 49) (Supplementary Table S2).

By NCBI blast, the *cps* gene clusters of *Klebsiella* spp. K31, K47, K61 and K63 were almost identical (>96% DNA identity) to those of *Escherichia coli* 5-172-05_S1_C3 (JOQS01000075.1), *Escherichia coli* HS (CP000802), *Escherichia coli* MS 85-1 (ADWQ01000010.1) and *Escherichia coli* KTE222 (ASUP01000016.1), respectively. Similarly, previous studies reported that *E. coli* and *Klebsiella* spp. possess highly similar *cps* sequences^23^,^31^. The *cps* sequences of *Klebsiella* spp. K4 also share high similarity (99% DNA identity) with those of *Serratia* spp. (AEQT01000901).

### General and atypical features of the *cps* locus in 79 capsular types of *Klebsiella* spp

The commonality of genetic features of the *Klebsiella* spp. *cps* loci has been revealed in previous studies^18^,^28^. Conserved genetic organization at the 5′ end of the *cps* locus extends from *galF* through *cpsACP, wzi, wza, wzb* and *wzc* and at the 3′ end of *cps* locus from *gnd* to *ugd*. The *wzc*-*gnd* region which usually contains genes encoding GTs, flippase (*wzx*), polymerase (*wzy*) and modifying enzymes (acetyltransferase, pyruvyl transferase, etc.) varies among different capsular types^18^. The *gnd*-*ugd* region possibly is composed of genes involved in GDP-D-mannose synthesis (*manB* and *manC*) or dTDP-L-rhamnose synthesis (*rmlA, rmlB, rmlC* and *rmlD*)^28^. Analysis of the 79 *cps* gene clusters from *Klebsiella* spp. indicated that these general features were observed in most capsular types, meanwhile, some notably uncommon features were characterized as well.

**Figure 1:**
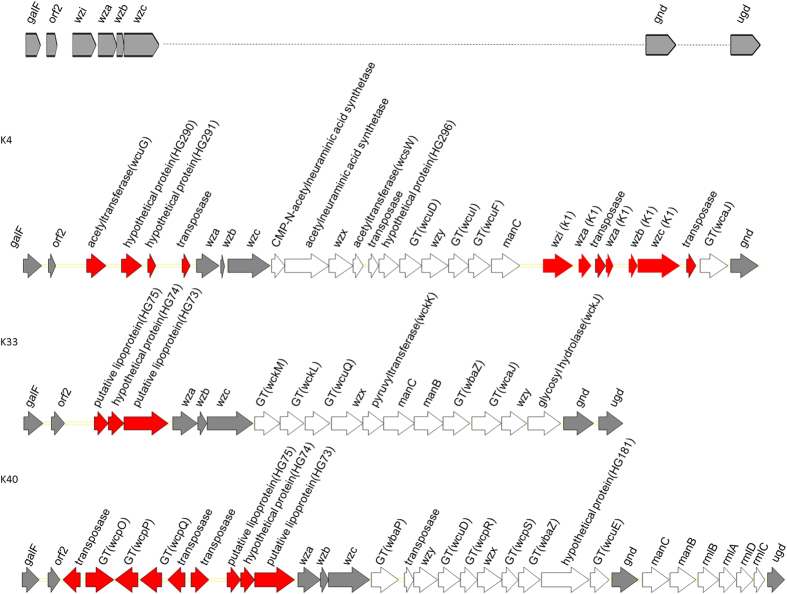
Genetic alignment of the K4, K33 and K40 *cps* gene cluster. Open reading frames (ORFs) are shown as arrows. The upper panel indicates conserved genetic organization of *cps* gene cluster. Atypical gene contents are marked in red color. GT, glycosyltransferase.

In K4, *cpsACP* was not followed by a *wzi* gene; instead, one acetyltransferase gene, two potassium/proton antiporter genes and a transposase gene were located between *cpsACP* and *wza* (Fig. 1). Similarly, the *wzi* gene was absent in K33 and K40 *cps* loci; instead, three hypothetical proteins were identified in the region of K33 (Fig. 1), and three GT genes, three genes encoding hypothetical proteins and three transposases genes were located in the region of K40 (Fig. 1). Besides, two *gnd* genes were found in the K41 *cps* region and most interestingly, the K4 *cps* was composed of a *wza*-*wzb*-*wzc* region and an additional *wzi*-*wza*-*wzb*-*wzc* region (*wza* was interrupted by a transposase gene). The additional three genes and the upstream *wzi* gene showed high DNA sequence identity with those of K1 (99% for each gene), indicating that K4 *cps* included several K1 *cps* genes (Fig. 1). Another atypical feature is that no *wzx*-like gene was found in capsular types K11 and K34 and no *wzy*-like gene was found in capsular types K29 and K50.

In addition, we further examined the correlation between the sugar composition and presence/absence of related *cps* genes. Of the 79 documented capsular types, to our best knowledge, 74 capsule structures are publicly available (the chemical structure are unavailable for the five types, K29, K42, K65, KN1 and KN2) (references were provided in Supplementary Table S3). Sugars found in different K-types are mannose for 37 types, fucose for 6 types, rhamnose for 28 types, and galactofuranose for 3 types. Among the 37 types with mannose as a structural unit, genes for GDP-D-mannose synthesis (*manB* and *manC*) were found in their *cps* regions, except in K4 (with *manC* only) and in K50 (both absent). Moreover, even there is no mannose incorporated into their capsule structures, capsular types K1, K16, K54, K58 and K63 harbored *manCB* genes. As the five types were known to use fucose as one of the components of their capsules and GDP-L-fucose is converted from GDP-D-mannose, mannose would be an intermediate rather than the final product incorporated into capsule. The six capsular types (K1, K6, K16, K54, K58, K63) that contain fucose as a structural unit possessed both *gmd* (gene encodes GDP-D-mannose 4, 6-dehydratase) and *wcaG* (a nucleotide sugar epimerase/dehydratase with bifunctional activity: GDP-4-dehydro-6-deoxy-D-mannose epimerase and GDP-4-dehydro-6-L-deoxygalactose reductase) genes, which are responsible for conversion from GDP-D-mannose to GDP-L fucose^32^. Conversely, the types with capsules that do not contain fucose lacked both *gmd* and *wcaG* in their *cps* regions.

The *rmlA, rmlB, rmlC* and *rmlD* genes are known to be responsible for dTDP-L-rhamnose synthesis^33^,^34^, and the four genes were usually found together, with the exception of the K65 *cps* region, which contained only the *rmlA, rmlC* and *rmlD* genes and not *rmlB*. From the resolved CPS structure, we found that the presence/absence of rhamnose in repeat units was perfectly correlated with presence/absence of *rmlBADC* genes. Galactofuranose was found in K12, K14 and K41, consistent with the presence of *glf* genes (encoding UDP-galactopyranose mutase, which catalyzes the conversion of UDP-galactopyranose into UDP-galactofuranose^35^,^36^).

With respect to the correlation between capsule modifications and modifying enzymes (acetyltransferases and pyruvyltransferases), 12 types exhibited acetylated capsules and 10 of them carried genes encoding acetyltransferases (the two exceptions are K33 and K59) (Supplementary Table S3). Sixty-two types express capsule without acetylation, but genes encoding acetyltransferases were found in 19 types. Twenty-eight types have pyruvylated capsules and contained genes that encoded pyruvyltransferases in their *cps* regions except for K11 (Supplementary Table S3). Forty-six types express capsule without pyruvylation, but gene for adding pyruvyl groups were found in 4 types (K8, K22, K37 and K66).

### Homology group (HG) assignment of *cps* genes

We used the TribeMCL program to assemble 1,515 predicted proteins into 361 HGs. The clustering result showed that 143 of the 361 HGs (40%) contained 2 to 81 members each, and the remainders formed 218 single-member HGs (Supplementary Table S2).

The products of *galF, wzi, wza, wzb, wzc, gnd, wcaJ, wbaP, manC, manB, rmlA, rmlB, rmlC* and *rmlD* fell into a single HG, suggesting these proteins were conserved among different capsular types. In contrast, non-initial GTs, Wzy polymerases and Wzx flippases were clustered into 142, 56 and 28 different HGs, respectively, indicating they were diverse in various types. Intriguingly, proteins for capsule modification (acetyltransferases and pyruvyltransferases) also classified into multiple groups (26 and 16 HGs, respectively), suggesting different modifying enzymes were needed for distinct capsule structures.

### Applications of *cps*-PCR genotyping

Due to the limitations of capsular serotyping, polymerase chain reaction-based genotyping of the capsular polysaccharide synthesis region, *cps*-PCR genotyping, was developed based on available *cps* sequences to detect specific *cps* genes in some capsular types of *Klebsiella* spp.^10^,^12^,^18^,^21^,^23^. Because *cps*-PCR genotyping is a rapid and accurate method for detecting the *cps* genotype, the availability of *cps* sequences in all 79 types will be very useful for discriminating capsular types based on capsular type-specific genes. According to the results of the protein clustering, non-initial GTs, Wzx and Wzy were specific to distinct capsular types, indicating these genes could be selected for genotyping. Because more than one non-initial GT gene was present in a given type, it would be easier to choose *wzx* or *wzy* as typing genes. In addition, since the 77 Wzy were classified into 56 HGs compared to the 77 Wzx categorized into 28 HGs, *wzy* exhibits more diversity than *wzx* in different types. There were 78 Wzi clustered into 4 HGs, suggesting that the *wzi* was less discriminatory than *wzx* or *wzy*. Therefore, *wzy* would be most specific for capsular PCR genotyping. We further analyzed the amino acid and DNA sequence identity of the *wzy* genes that were grouped into the same HG groups. Most of the Wzy proteins shared <60% amino acid sequences identity even within a single HG group and shared DNA sequence similarity with <600 bp matching sequences over ~1.2 kb gene length except K22 vs. K37, K12 vs. K41, K2 vs. K13, K74 vs. K80, K79 vs. KN1 and K30 vs. K69 (Table 1). Previous studies have documented that K22 and K37 possess identical *wzy* genes and are only distinguishable by the acetyltransferase encoding genes^23^. For the types exhibited high similarity (>600 bp matching sequences over ~1.2 kb gene length), primers should be designed according to the variable region of their *wzy* genes; alternatively, other *cps* genes can be used for differentiating these types. Another limitation is the inapplicability in the capsular types lacking *wzy*-like genes (K29 and K50).

### Enzymes for synthesis of capsular repeat unit

WbaP and WcaJ regarded as initial GT for capsule synthesis are UDP-hexose transferase enzymes that transfer galactose-1-phosphate and glucose-1-phosphate, respectively, to undecaprenol phosphate^28^,^37^. Additional transferases (non-initial GT) further add sugars to form repeat units^29^,^38^ and polymerase enzyme, Wzy, subsequently assemble the lipid-linked repeat units^29^. We found that either *wbaP* or *wcaJ* were present in the 79 *cps* loci, and the clustering results showed that the initial GTs (WbaP and WcaJ) were assembled into a single group each, implying they were conserved among different types. Furthermore, a perfect correlation was observed in the 74 types with available capsule structures, that is, *wbaP* genes co-exist with the presence of galactose in the repeat unit, and *wcaJ* co-exist with the presence of glucose. Moreover, possible polymerization linkage of the repeat unit can be predicted based on which type of initial GT they possess. For example, the presence of *wcaJ* indicates that glucose is the initial sugar of K1 capsular repeat units, therefore, the polymerization linkage of K1 capsular repeat units could be β-D-Glcp(1 → 4)β-D-GlcpA according to reported chemical structure of its capsule^39^ and K1 Wzy (MagA) is supposed to be responsible for the linkage formation. In addition, K12 and K41 which share 82% amino acid sequences identity in Wzy seem to have the same predicted polymerization linkage for their capsular repeat units, i.e., α-D-Galp(1 → 2)β-D-galf^40^,^41^.

The clustering results showed that 318 non-initial GTs were clustered into 142 different HGs, which provide some information on the possible functions of the GTs. For example, one HG (HG20) contains 14 GTs from K3, K7, K21, K24, K26, K28, K29, K39, K40, K43, K53, K65, K74 and K80 (the GTs show 37-64% amino acid identity to their members). Based on the available CPS structures (except for K29 and K65), 10 of the 12 types (K3, K7, K21, K24, K26, K28, K43, K53, K74 and K80) share the same linkage α-D-Manp(1 → 2)α-D-Manp. Therefore, we suggested that these GTs grouped into the same HG (named as WcuE) probably has catalytic activity for the specific sugar linkage. Accordingly, the relationship of GTs and CPS structures lays the foundations for understanding the putative functions of different GTs.

### Capsular types with related *cps* genes and similar capsule structure

According to the protein clustering results, 9 pairs of capsular types (K1 and K58, K2 and K13, K12 and K41, K14 and K64, K10 and K61, K30 and K69, K33 and K35, K74 and K80, and K57 and K68) have 5 or more genes that located within the variable region (*wzc*-*ugd*, excluding *man* and *rml* genes) shared similarity (clustered into the same HG). Therefore, we compared the CPS structures of these capsular types and indicated correlations between genes and products. Below are 6 examples with clearer implications (others are described in Supplementary Fig. S1a–S1c):

#### K1 and K58

**Figure 2:**
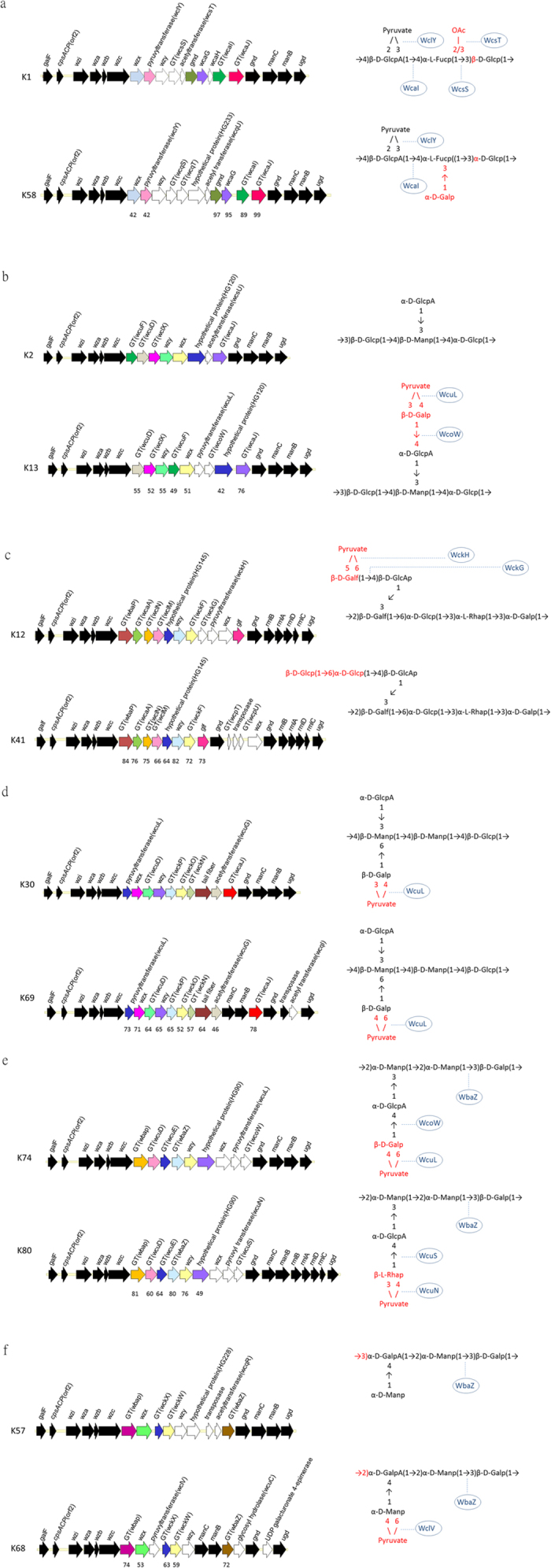
Comparison of *cps* gene clusters and capsule structures in capsular types with similar *cps* gene content. Open reading frames (ORFs) are shown as arrows. Conserved genes, *man* genes, *rml* genes or transposases are shown in black. Other gene products that were clustered into the same HGs are shown in same colors, and the amino acid similarities (%) are indicated below the ORFs. Genes only present in either of the two types are shown in white. GT, glycosyltransferase. Enzymes most likely involved in linkage formation are indicated along with their capsular structures. The differences of capsule structures from two types are shown in red. a, K1 and K58; b, K2 and K13; c, K12 and K41; d, K30 and K69; e, K74 and K80; f, K57 and K68.

The same linkage of β-D-GlcpA(1 → 4)α-L-Fucp was found in the capsular repeat units of K1 and K58^39^,^42^, thus, we suggest that the fucosyl transferase WcaI present in both types is responsible for the synthesis of this linkage. Moreover, The specific GT in K1, wcsS, most likely accounts for the linkage α-L-Fucp((1 → 3)β-D-Glcp; whereas the two GTs in K58, WcqS and WcqT, likely accounts for the α-L-Fucp((1 → 3)α-D-Glcp linkage or the side chain synthesis (Fig. 2a and Supplementary Table S4). As for the similarity of the genetic organization of *cps* clusters, it is not surprising that serological cross reactions are reported between the two types^24^.

#### K2 and K13

K2 and K13, which are known to cross-react by serotyping^43^,^44^, share similar capsule structures that only differ in the side chain, i.e., α-D-GlcpA(1 → 3)β-D-Manp in K2 and 3, 4-Pyr-β-D-Galp(1 → 4)α-D-GlcpA(1 → 3)β-D-Manp in K13^45^. The pyruvyl transferase (WcuL) and the GT (WcoW) present only in K13 but not in K2 may contribute to the addition of the pyruvyl group and the synthesis of the linkage β-D-Galp(1 → 4)α-D-GlcpA, respectively (Fig. 2b and Supplementary Table S4). The function of WcoW was also evidenced by the co-existence of WcoW and the linkage in K74^46^. In addition, we also found that K2 has an acetyltransferase-encoding gene; however, the previously reported K2 capsule structure is not acetylated^45^.

#### K12 and K41

Serological cross-reactions between K12 and K41 are known to occur^47^,^48^. The two capsular types exhibit the same repeat unit but distinct side branches^40^,^41^. The side chain of K12 was determined to be 5, 6-Pyr-β-D-Galf(1 → 4)β-D-GlcpA(1 → 3)β-D-Galf and that of K41 is β-D-Glcp(1 → 6)α-D-Glcp((1 → 4)β-D-GlcpA(1 → 3)β-D-Galf. A GT (wckG) and a pyruvyl transferase (wckH) were found only in K12, suggesting that these are the key enzymes involved in the synthesis of β-D-Galf(1 → 4)β-D-GlcpA and pyruvylation, respectively (Fig. 2c and Supplementary Table S4). And The two GTs (WcpT and WcpU) in K41 are likely involved in the synthesis of β-D-Glcp(1 → 6)α-D-Glcp((1 → 4)β-D-GlcpA.

#### K30 and K69

Even no cross-reaction has been reported between K30 and K69, the capsule structures of the two types are almost identical with the exception of the linkage between β-D-Galp and the pyruvyl group^49^,^50^. The *cps* regions of the two types were also highly similar (Fig. 2d and Supplementary Table S4). With the major difference between these two strains being pyruvylation, the pyruvyltransferases from K30 and K69 which shared 73% amino acid identity (named as WcuL) could catalyze both pyruvylation linkages or the dissimilarity of the two proteins is critical for their specificity.

#### K74 and K80

K74 and K80 exhibit similar capsule structures^46^,^51^ and *cps* genes but do not show serological cross-reactivity. The differences between these two types reside within the side chains: 4, 6-Pyr-β-D-Galp(1 → 4)α-D-GlcpA(1 → 3)α-D-Manp and 3, 4-Pyr-β-L-Rhap(1 → 4)α-D-GlcpA(1 → 3)α-D-Manp in K74 and K80, respectively. Comparing the gene content of the K74 and K80 *cps* loci, genes for rhamnose synthesis (*rmlABCD*) were found in K80 but not in K74, which is consistent with the use of rhamnose in the side chain of K80 (Fig. 2e and Supplementary Table S4). Moreover, K74 and K80 each possess a unique pyruvyl transferase and a GT, suggesting that WcuL is involved in the synthesis of 4, 6-Pyr-β-D-Galp (the predicted function of WcuL is the same as what we proposed for the K69 structure); WcoW is involved in the synthesis of β-D-Galp(1 → 4)α-D-GlcpA (the predicted function of WcoW is the same as what we proposed for the K13 structure); WcuN likely accounts for the synthesis of 3, 4-Pyr-β-L-Rhap; and WcuS likely accounts for the synthesis of β-L-Rhap(1 → 4)α-D-GlcpA. Moreover, because WbaZ is known to catalyze the α-D-Manp-(1 → 3)β-D-Galp glycosidic linkage^38^, WcuD and WcuE were presumably responsible for the synthesis of the rest of linkages, i.e., α-D-Manp(1 → 2)α-D-Manp or α-D-GlcpA(1 → 3)α-D-Manp.

#### K57 and K68

K57 and K68 do not exhibit serological cross-reactivity but showed similarity in CPS structures^45^,^52^ and *cps* genes. The GT WbaZ which is known to form the α-D-Manp-(1 → 3)β-D-Galp disaccharide backbone is present in both strains. The pyruvylation of the capsule in K68 is also indicated by the presence of a pyruvyl transferase gene within its *cps* locus. In addition, the GTs WckX and WckW from K57 exhibited similarity with those of K68, implicating that the two proteins are responsible for the common linkages of the two types: α-D-Manp-(1 → 4)α-D-GalpA on the side chains and the α-D-GalpA(1 → 2)α-D-Manp linkage in the backbone (Fig. 2f and Supplementary Table S4).

## Discussion

A notable finding of this study is that K4 *cps* region was mosaicked with K1 *cps* genes, implicating *cps* gene could shuffle within *Klebsiella* spp. Therefore, lateral gene transfers of *cps* loci either intra- or inter- species could frequently occur as capsule switching was evidenced by a recent study which revealed that high number of distinct *cps* variants within *K. pneumoniae* clonal group CG258 were caused by extensive recombination events between distinct *cps*^53^.

We also found that some common *cps* genes were absent or truncated in a few types. Chances are that gene homologues in other locations of genome could compensate the functions, or mechanisms other than the typical group 1 system could be involved in capsule biosynthesis for these types. Another possibility could be the actual loss of this gene function, such as the K50 capsular type reference strain has been observed to be non-capsulated^25^.

Another notable feature of the *cps* loci is the existence of genes encoding transposases or phage-related proteins, which may be evidence that transposition and horizontal gene transfer has occurred within *cps* regions. Some chromosomal rearrangements associated with transposition events may lead to gene loss. A previous study found that the *wzb*-*wzc* locus of the *cps* region was replaced by transposase genes in K15 and K50, which resulted in capsule deficiency^23^. In some cases, transposases most likely modify the *cps* region instead of disrupting it. For example, it has been documented that the *cps* gene clusters of *Streptococcus pneumoniae* serogroup 12 and serotypes 44 and 46 only differ in the presence of transposase genes^54^. Although we did not find any documented capsular types of *Klebsiella* spp. that differ only in transposases or transposons, we hypothesize that other strains will likely display a subtype or new type by transposase or transposon integration.

*cps* genotyping based on *wzi* sequencing has been used for discriminating the capsular types of *Klebsiella* spp.^10^. *wzy* genes were highly variable while *wzi* genes were relatively conserved. *wzy* PCR genotyping needed specific primers from each already resolved sequences, however, it was more specific and no sequencing was necessary. In contrast, *wzi* genotyping could use relatively conserved sequences as primers, but it needed PCR and sequencing of PCR products to obtain final results. Both methods would encounter difficulties in some capsular types unless full *cps* sequences available. *wzc* genotyping^25^ was similar to *wzi* PCR and sequencing. However, it can differentiate much more reported genotypes than *wzi*. Therefore, *wzy* PCR would be preferable to rapid identify a specific genotype while *wzc* PCR with sequencing would be best to test isolates with unknown type prevalence.

Comparative analysis of different capsular types showed their relatedness, and the genetic differences (presence or absence of genes, sequence changes and gene truncation etc.) can be linked to the various structures of the expressed capsules. Our results indicated that some types exhibit similar capsule structures because of the high similarity in their *cps* regions. In terms of serological reactions, some of the capsular types that share related *cps* genes are known to cross-react by serotyping (K1 vs. K58; K2 vs. K13; K12 vs. K41), indicating that anti-sera recognize their common structures; other strains do not exhibit cross-reactivity despite sharing very similar structures (K30 vs. K69; K74 vs. K80; K57 vs. K68), suggesting that distinct epitopes are crucial for serological differentiation. In addition, putative functions of *cps* genes were also indicated according to the presence of specific genes and unique linkages. The existence of genes for capsule modifications in *cps* region also revealed the possibility of presence of undefined capsule modifications in certain types. Besides, we also provide some evidence of sugar composition in types with unknown CPS structure. Therefore, as all *cps* gene clusters from different capsular types of *Klebsiella* spp. have been resolved, the functions of genes involved in capsule synthesis will be much clear.

In conclusion, the available *cps* sequences and comparative analysis of various capsular types has an impact on understanding of the functions of *cps* genes and provides complete information on the relatedness of different capsular types through evolutionary history. Furthermore, these data are an important basis for the application of capsular genotyping as well as new type identification in *Klebsiella* spp.

## Methods

### Bacterial strains

A total of 77 K-serotype *Klebsiella* spp. reference strains were purchased from Statens Serum Institute, Copenhagen, Denmark. Two additional strains with novel type KN1 and KN2 capsules identified in our laboratory were also included^12^,^13^.

### Sequencing of *cps* loci

We amplified the *cps* loci from *Klebsiella* spp. strains using multiple pairs of conserved primers as previously described^12^,^55^ (Supplementary Table S5 and Supplementary Fig. S2). PCR amplifications were performed with the Long and Accurate PCR system, and the cycling programs were used in accordance with previously described procedures^12^. The PCR amplicons were subjected to sequencing by high-throughput sequencing (Yang-Ming Genome Research Center) using the Illumina/Solexa GAII sequencing platform. When PCR amplifications failed, *cps* sequences were obtained by previously described inverse PCR and DNA sequencing methods^56^ based on the available *wzc* sequences of these types^23^. The *cps* sequences (approximately 20–30 kb) were deposited in Genbank (Accession Numbers are shown in Supplementary Table S1).

### Gene annotation and homology group (HG) assignment

Coding sequences were predicted by vector NTI and annotated by NCBI-protein blast. Predicted proteins were classified into HGs using the TribeMCL algorithm (Centre for Mathematics and Computer Science and EMBL-EBI)^57^ with a cut-off of 1e^−50^. Gene names were assigned for *cps* genes encoding GTs, acetyltransferases and pyruvyltransferases in accordance with the Bacterial Polysaccharide Gene Database^58^ if they had not been given names previously. Proteins within the same HGs were given the same name, and hypothetical proteins with uncertain roles in capsule synthesis were given names according to the number of HGs. The polymerases (Wzy) that fell into multiple HGs were each assigned a number to indicate the different groups.

## Additional Information

**How to cite this article**: Pan, Y.-J. *et al.* Genetic analysis of capsular polysaccharide synthesis gene clusters in 79 capsular types of *Klebsiella* spp. *Sci. Rep.* **5**, 15573; doi: 10.1038/srep15573 (2015).

## Supplementary Material

Supplementary Information

## Figures and Tables

**Table 1:** Amino acid and DNA sequences identity of the members in Wzy HG groups.

**Wzy group**	**Capsular types of the members**	**Types for comparison**	**Sequences identity (%)**^a^	
			**Amino acid**	**DNA**
1(HG46)	K52, K53, K81	K52 vs. K81	144/376(38%)	193/292(66%)
		K52 vs. K53	133/377(35%)	—
		K81 vs. K53	134/377(36%)	—
2(HG49)	K42, K59, K65	K59 vs. K65	167/415(40%)	—
		K59 vs. K42	152/388(39%)	—
		K65 vs. K42	156/388(40%)	—
3(HG50)	K36, K48, K67	K48 vs. K36	127/303(42%)	146/214(68%)
		K48 vs. K67	110/296(37%)	—
		K36 vs. K67	118/295(38%)	—
4(HG51)	K27, K38, KN2	K27 vs. K38	128/409(31%)	—
		K27 vs. KN2	145/403(36%)	—
		K38 vs. KN2	143/423(34%)	—
5(HG56)	K22, K25, K37	K22 vs. K25	194/380(51%)	540/842(64%)
		K22 vs. K37	381/381(100%)	1143/1143(100%)
6(HG58)	K3, K24, K28	K3 vs. K24	179/373(48%)	173/269(64%)
		K3 vs. K28	167/394(42%)	—
		K24 vs. K28	145/394(37%)	—
7(HG80)	K30, K69	K30 vs. K69	269/412(65%)	819/1192(69%)
8(HG88)	K79, KN1	K79 vs. KN1	239/364(66%)	803/1167(69%)
9(HG91)	K74, K80	K74 vs. K80	308/407(76%)	831/1141(73%)
10(HG101)	K45, K72	K45 vs. K72	149/389(38%)	—
11(HG105)	K35, K46	K35 vs. K46	158/387(41%)	380/602(63%)
12(HG110)	K11, K82	K11 vs. K82	135/349(39%)	—
13(HG121)	K2, K13	K2 vs. K13	228/415(55%)	747/1180(63%)
14(HG140)	K14, K64	K14 vs. K64	201/397(51%)	241/357(68%)
15(HG125)	K12, K41	K12 vs. K41	324/393(82%)	894/1174(76%)

Note:

^a^the two sequences were aligned by NCBI blastp for amino acids analysis and NCBI blastn for nucleotide analysis; the ratio of identity indicates no. of matching nucleotides or amino acids/total no. of nucleotides or amino acids; — indicates < 100 bp nucleotides were matched.

## References

[b1] Abbot S. L. Klebsiella, Enterobacter, Citrobacter, Serratia, Plesiomonas, and other Enterobacteriaceae. In: Murray P. R., Baron E. J., Jorgensen J. H., Pfaller M. A., Yolken R. H., eds. Manual of clinical microbiology. 8Ch ed. Washington DC, American Society for Microbiology Press, 684–700 (2003).

[b2] Podschun R. & Ullmann U. Klebsiella spp. as nosocomial pathogens: epidemiology, taxonomy, typing methods, and pathogenicity factors. Clinical Microbiology Reviews 11, 589–603 (1998). 9767057 10.1128/cmr.11.4.589 PMC88898

[b3] Lin W. H. *et al.* Clinical and microbiological characteristics of Klebsiella pneumoniae isolates causing community-acquired urinary tract infections. Infection 38, 459–64 (2010). 20734217 10.1007/s15010-010-0049-5

[b4] Tsai F. C., Huang Y. T., Chang L. Y. & Wang J. T. Pyogenic liver abscess as endemic disease, Taiwan. Emerg Infect Dis 14, 1592–600 (2008). 18826824 10.3201/eid1410.071254 PMC2609891

[b5] Wang J. L. *et al.* Changing bacteriology of adult community-acquired lung abscess in Taiwan: Klebsiella pneumoniae versus anaerobes. Clinical infectious diseases: an official publication of the Infectious Diseases Society of America 40, 915–22 (2005). 15824979 10.1086/428574

[b6] Fang C. T. *et al.* Klebsiella pneumoniae genotype K1: an emerging pathogen that causes septic ocular or central nervous system complications from pyogenic liver abscess. Clinical infectious diseases: an official publication of the Infectious Diseases Society of America 45, 284–93 (2007). 17599305 10.1086/519262

[b7] Fung C. P. *et al.* A global emerging disease of Klebsiella pneumoniae liver abscess: is serotype K1 an important factor for complicated endophthalmitis? Gut 50, 420–4 (2002). 11839725 10.1136/gut.50.3.420 PMC1773126

[b8] Cortes G. *et al.* Molecular analysis of the contribution of the capsular polysaccharide and the lipopolysaccharide O side chain to the virulence of Klebsiella pneumoniae in a murine model of pneumonia. Infect Immun 70, 2583–90 (2002). 11953399 10.1128/IAI.70.5.2583-2590.2002 PMC127904

[b9] Mizuta K. *et al.* Virulence for mice of Klebsiella strains belonging to the O1 group: relationship to their capsular (K) types. Infect Immun 40, 56–61 (1983). 6187694 10.1128/iai.40.1.56-61.1983 PMC264817

[b10] Brisse S. *et al.* wzi Gene sequencing, a rapid method for determination of capsular type for Klebsiella strains. J Clin Microbiol 51, 4073–8 (2013). 24088853 10.1128/JCM.01924-13 PMC3838100

[b11] Ørskov I. & Fife-Asbury M. A. New Klebsiella capsular antigen K82 and the deletion of five of those previously assigned. International Journal of Systematic Bacteriology 27, 386–7 (1977).

[b12] Pan Y. J. *et al.* Capsular polysaccharide synthesis regions in Klebsiella pneumoniae serotype K57 and a new capsular serotype. Journal of Clinical Microbiology 46, 2231–40 (2008). 18508935 10.1128/JCM.01716-07 PMC2446917

[b13] Hsu C. R., Lin T. L., Pan Y. J., Hsieh P. F. & Wang J. T. Isolation of a bacteriophage specific for a new capsular type of Klebsiella pneumoniae and characterization of its polysaccharide depolymerase. PLoS One 8, e70092 (2013). 23936379 10.1371/journal.pone.0070092 PMC3732264

[b14] Julianelle L. A. A Biological Classification of Encapsulatus Pneumoniae (Friedlander’s Bacillus). J Exp Med 44, 113–28 (1926). 19869169 10.1084/jem.44.1.113 PMC2180261

[b15] Fung C. P. *et al.* A 5-year study of the seroepidemiology of Klebsiella pneumoniae: high prevalence of capsular serotype K1 in Taiwan and implication for vaccine efficacy. The Journal of infectious diseases 181, 2075–9 (2000). 10837197 10.1086/315488

[b16] Tsay R. W., Siu L. K., Fung C. P. & Chang F. Y. Characteristics of bacteremia between community-acquired and nosocomial Klebsiella pneumoniae infection: risk factor for mortality and the impact of capsular serotypes as a herald for community-acquired infection. Arch Intern Med 162, 1021–7 (2002). 11996612 10.1001/archinte.162.9.1021

[b17] Jenney A. W. *et al.* Seroepidemiology of Klebsiella pneumoniae in an Australian Tertiary Hospital and its implications for vaccine development. Journal of Clinical Microbiology 44, 102–7 (2006). 16390956 10.1128/JCM.44.1.102-107.2006 PMC1351949

[b18] Chuang Y. P., Fang C. T., Lai S. Y., Chang S. C. & Wang J. T. Genetic determinants of capsular serotype K1 of Klebsiella pneumoniae causing primary pyogenic liver abscess. Journal of Infectious Diseases 193, 645–54 (2006). 16453259 10.1086/499968

[b19] Fang F. C., Sandler N. & Libby S. J. Liver abscess caused by magA+ Klebsiella pneumoniae in North America. Journal of Clinical Microbiology 43, 991–2 (2005). 15695726 10.1128/JCM.43.2.991-992.2005 PMC548117

[b20] Struve C., Bojer M., Nielsen E. M., Hansen D. S. & Krogfelt K. A. Investigation of the putative virulence gene magA in a worldwide collection of 495 Klebsiella isolates: magA is restricted to the gene cluster of Klebsiella pneumoniae capsule serotype K1. J Med Microbiol 54, 1111–3 (2005). 16192445 10.1099/jmm.0.46165-0

[b21] Fang C. T. *et al.* Klebsiella pneumoniae genotype K1: an emerging pathogen that causes septic ocular or central nervous system complications from pyogenic liver abscess. Clin Infect Dis 45, 284–93 (2007). 17599305 10.1086/519262

[b22] Yu W. L. *et al.* Polymerase chain reaction analysis for detecting capsule serotypes K1 and K2 of Klebsiella pneumoniae causing abscesses of the liver and other sites. J Infect Dis 195, 1235–6; author reply 1236 (2007). 17357063 10.1086/512686

[b23] Pan Y. J. *et al.* Capsular Types of Klebsiella pneumoniae Revisited by wzc Sequencing. PLoS ONE 8, e80670 (2013). 24349011 10.1371/journal.pone.0080670 PMC3857182

[b24] Brisse S., Issenhuth-Jeanjean S. & Grimont P. A. Molecular serotyping of Klebsiella species isolates by restriction of the amplified capsular antigen gene cluster. J Clin Microbiol 42, 3388–98 (2004). 15297473 10.1128/JCM.42.8.3388-3398.2004 PMC497587

[b25] Pan Y. J. *et al.* Capsular types of Klebsiella pneumoniae revisited by wzc sequencing. PLoS One 8, e80670 (2013). 24349011 10.1371/journal.pone.0080670 PMC3857182

[b26] Arakawa Y. *et al.* Genomic organization of the Klebsiella pneumoniae cps region responsible for serotype K2 capsular polysaccharide synthesis in the virulent strain Chedid. Journal of Bacteriology 177, 1788–96 (1995). 7896702 10.1128/jb.177.7.1788-1796.1995 PMC176807

[b27] Rahn A., Drummelsmith J. & Whitfield C. Conserved organization in the cps gene clusters for expression of Escherichia coli group 1 K antigens: relationship to the colanic acid biosynthesis locus and the cps genes from Klebsiella pneumoniae. J Bacteriol 181, 2307–13 (1999). 10094716 10.1128/jb.181.7.2307-2313.1999 PMC93651

[b28] Shu H. Y. *et al.* Genetic diversity of capsular polysaccharide biosynthesis in Klebsiella pneumoniae clinical isolates. Microbiology 155, 4170–83 (2009). 19744990 10.1099/mic.0.029017-0

[b29] Whitfield C. Biosynthesis and assembly of capsular polysaccharides in Escherichia coli. Annu Rev Biochem 75, 39–68 (2006). 16756484 10.1146/annurev.biochem.75.103004.142545

[b30] Whitfield C. & Roberts I. S. Structure, assembly and regulation of expression of capsules in Escherichia coli. Molecular microbiology 31, 1307–19 (1999). 10200953 10.1046/j.1365-2958.1999.01276.x

[b31] Rahn A., Drummelsmith J. & Whitfield C. Conserved organization in the cps gene clusters for expression of Escherichia coli group 1 K antigens: relationship to the colanic acid biosynthesis locus and the cps genes from Klebsiella pneumoniae. Journal of Bacteriology 181, 2307–13 (1999). 10094716 10.1128/jb.181.7.2307-2313.1999 PMC93651

[b32] Albermann C., Distler J. & Piepersberg W. Preparative synthesis of GDP-beta-L-fucose by recombinant enzymes from enterobacterial sources. Glycobiology 10, 875–81 (2000). 10988249 10.1093/glycob/10.9.875

[b33] Giraud M. F. & Naismith J. H. The rhamnose pathway. Curr Opin Struct Biol 10, 687–96 (2000). 11114506 10.1016/s0959-440x(00)00145-7

[b34] Koplin R., Wang G., Hotte B., Priefer U. B. & Puhler A. A 3.9-kb DNA region of Xanthomonas campestris pv. campestris that is necessary for lipopolysaccharide production encodes a set of enzymes involved in the synthesis of dTDP-rhamnose. J Bacteriol 175, 7786–92 (1993). 8253667 10.1128/jb.175.24.7786-7792.1993 PMC206953

[b35] Nassau P. M. *et al.* Galactofuranose biosynthesis in Escherichia coli K-12: identification and cloning of UDP-galactopyranose mutase. J Bacteriol 178, 1047–52 (1996). 8576037 10.1128/jb.178.4.1047-1052.1996 PMC177764

[b36] Lee R. *et al.* Enzymatic synthesis of UDP-galactofuranose and an assay for UDP-galactopyranose mutase based on high-performance liquid chromatography. Anal Biochem 242, 1–7 (1996). 8923956 10.1006/abio.1996.0419

[b37] Liu D., Haase A. M., Lindqvist L., Lindberg A. A. & Reeves P. R. Glycosyl transferases of O-antigen biosynthesis in Salmonella enterica: identification and characterization of transferase genes of groups B, C2, and E1. J Bacteriol 175, 3408–13 (1993). 7684736 10.1128/jb.175.11.3408-3413.1993 PMC204739

[b38] Drummelsmith J. & Whitfield C. Gene products required for surface expression of the capsular form of the group 1 K antigen in Escherichia coli (O9a:K30). Mol Microbiol 31, 1321–32 (1999). 10200954 10.1046/j.1365-2958.1999.01277.x

[b39] Ho J. Y. *et al.* Functions of some capsular polysaccharide biosynthetic genes in Klebsiella pneumoniae NTUH K-2044. PLoS One 6, e21664 (2011). 21765903 10.1371/journal.pone.0021664 PMC3134468

[b40] Beurret M., Joseleau J. P., Vignon M., Dutton G. G. & Savage A. V. Proof of the occurrence of 5,6-O-(1-carboxyethylidene)-D-galactofuranose units in the capsular polysaccharide of Klebsiella K12. Carbohydr Res 189, 247–60 (1989). 2550127 10.1016/0008-6215(89)84101-1

[b41] Beurret M., Joseleau J. P., Dutton G. G. & Savage A. V. Homologous and heterologous reactions of bacteriophages phi 41 and phi 12 on the capsular polysaccharides from Klebsiella K41 and K12. Carbohydr Res 189, 237–46 (1989). 2776136 10.1016/0008-6215(89)84100-x

[b42] Dutton G. S. & Savage A. V. Structural investigation of the capsular polysaccharide of klebsiella serotype K58. Carbohydr Res 84, 297–305 (1980). 10.1016/0008-6215(80)90011-7 7353210

[b43] Pieroni P., Rennie R. P., Ziola B. & Deneer H. G. The use of bacteriophages to differentiate serologically cross-reactive isolates of Klebsiella pneumoniae. J Med Microbiol 41, 423–9 (1994). 7966220 10.1099/00222615-41-6-423

[b44] Ørskov I. & Ørskov F. Serotyping of Klebsiella. Methods Microbiol. 14, 143–64 (1984).

[b45] Geyer H., Himmelspach K., Kwiatkowski B., Schlecht S. & Stirm S. Degradation of bacterial surface carbohydrates by virus-associated enzymes. Pure & Appl. Chem. 55, 637–53 (1983).

[b46] Dutton G. S. & Paulin M. Structure of the capsular polysaccharide of Klebsiella serotype K74. Carbohydr Res 87, 119–27 (1980). 7438137 10.1016/s0008-6215(00)85196-4

[b47] Riser E., Noone P. & Poulton T. A. A new serotyping method for Klebsiella species: development of the technique. J Clin Pathol 29, 296–304 (1976). 777042 10.1136/jcp.29.4.296 PMC476050

[b48] Murcia A. & Rubin S. J. Reproducibility of an indirect immunofluorescent-antibody technique for capsular serotyping of Klebsiella pneumoniae. J Clin Microbiol 9, 208–13 (1979). 372225 10.1128/jcm.9.2.208-213.1979 PMC272993

[b49] Lindberg B., Lindh F., Lonngren J. & Sutherland I. W. Structural studies of the capsular polysaccharide of Klebsiella type 30. Carbohydr Res 76, 281–4 (1979). 526959 10.1016/0008-6215(79)80031-2

[b50] Hackland P. L., Parolis H. & Parolis L. A. A structural investigation of the capsular polysaccharide of Klebsiella K69. Carbohydr Res 172, 209–16 (1988). 3370648 10.1016/s0008-6215(00)90855-3

[b51] Dutton G. S. & Karunaratne D. Structural investigation of the capsular polysaccharide of Klebsiella serotype K80. Carbohydr Res 134, 103–14 (1984).

[b52] Dutton G. G., Parolis H. & Parolis L. A. The structural elucidation of the capsular polysaccharide of Klebsiella K68. Carbohydr Res 152, 249–59 (1986). 3768910 10.1016/s0008-6215(00)90305-7

[b53] Wyres K. L. *et al.* Extensive capsule locus variation and large-scale genomic recombination within the Klebsiella pneumoniae clonal group 258. Genome Biol Evol (2015). 10.1093/gbe/evv062 PMC4453057 25861820

[b54] Bentley S. D. *et al.* Genetic analysis of the capsular biosynthetic locus from all 90 pneumococcal serotypes. PLoS Genet 2, e31 (2006). 16532061 10.1371/journal.pgen.0020031 PMC1391919

[b55] Brisse S., Issenhuth-Jeanjean S. & Grimont P. A. Molecular serotyping of Klebsiella species isolates by restriction of the amplified capsular antigen gene cluster. Journal of Clinical Microbiology 42, 3388–98 (2004). 15297473 10.1128/JCM.42.8.3388-3398.2004 PMC497587

[b56] Chang K. C., Yeh Y. C., Lin T. L. & Wang J. T. Identification of genes associated with natural competence in Helicobacter pylori by transposon shuttle random mutagenesis. Biochem Biophys Res Commun 288, 961–8 (2001). 11689003 10.1006/bbrc.2001.5877

[b57] Enright A. J., Van Dongen S. & Ouzounis C. A. An efficient algorithm for large-scale detection of protein families. Nucleic Acids Res 30, 1575–84 (2002). 11917018 10.1093/nar/30.7.1575 PMC101833

[b58] Reeves P. R. *et al.* Bacterial polysaccharide synthesis and gene nomenclature. Trends Microbiol 4, 495–503 (1996). 9004408 10.1016/s0966-842x(97)82912-5

