# Supplementary material for: Genetic analysis of capsular polysaccharide synthesis gene clusters in 79 capsular types of Klebsiella spp
Source: Sci Rep. 2015 Oct 23;5:15573. doi: 10.1038/srep15573 (PMC4616057; doi:10.1038/srep15573)
Supplement: Supplementary Information [file srep15573-s1.doc]

**Genetic analysis of capsular polysaccharide synthesis gene clusters in 79 capsular types of *Klebsiella* spp.**

Yi-Jiun Pan ^1a^, Tzu-Lung Lin^1a^, Chun-Tang Chen^1^, Yi-Yin Chen^1^, Pei-Fang Hsieh^1^, Chun-Ru Hsu^1^, Meng-Chuan Wu^1^ and Jin-Town Wang^1, 2*^

^1^Department of Microbiology, National Taiwan University College of Medicine, Taipei, Taiwan

^2^Department of Internal Medicine, National Taiwan University Hospital, Taipei, Taiwan

^*^correspondence: Dr. Jin-Town Wang

Department of Microbiology, National Taiwan University College of Medicine

1, Sec 1, Jen-Ai Rd., Taipei, Taiwan.

Tel: +886-2-23123456 ext 88292; fax: +886-2-23948718.

*E-mail*: [wangjt@ntu.edu.tw](mailto:wangjt@ntu.edu.tw)

^a^These two authors contribute equally in this work.


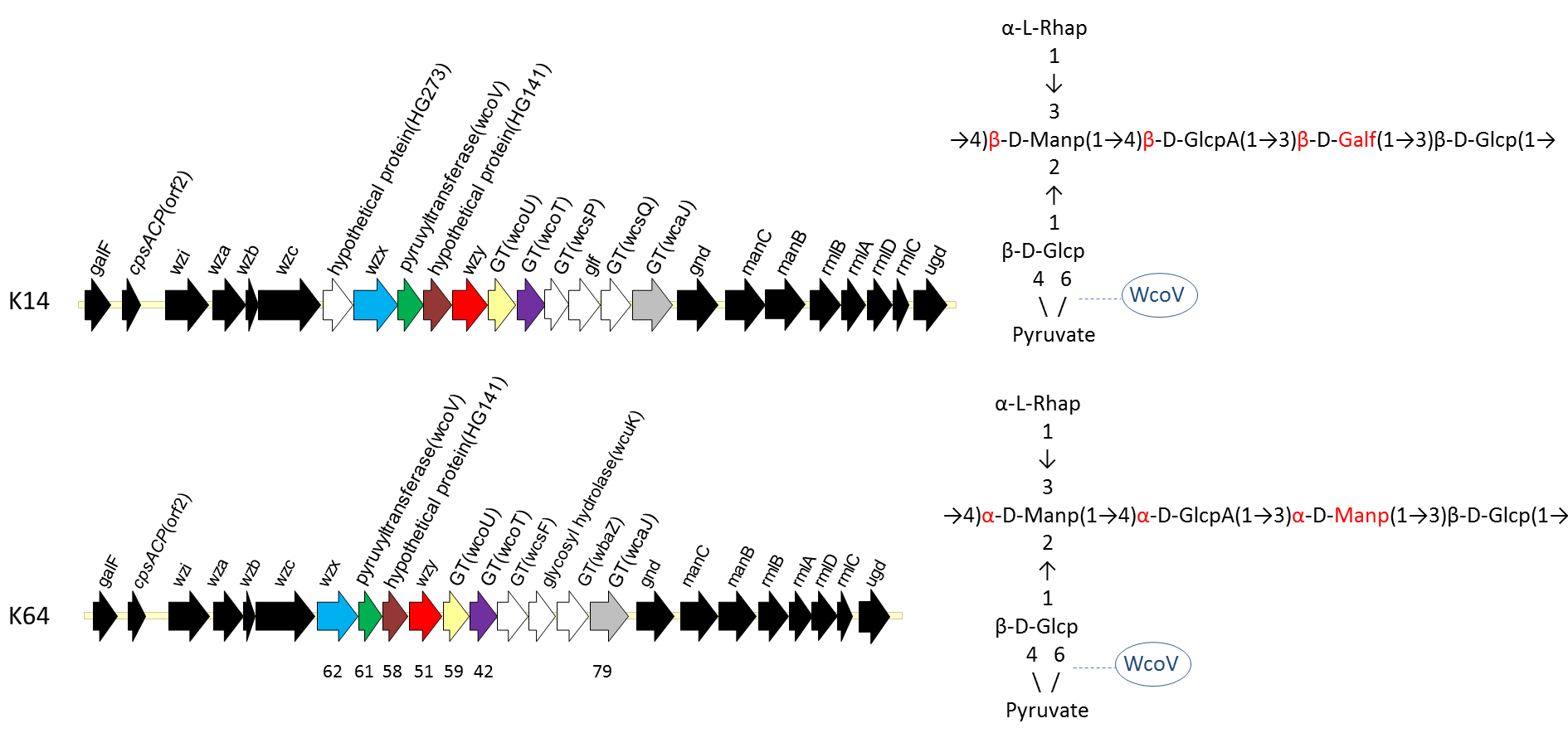


**Supplementary Figure S1a**. Comparison of *cps* gene clusters and capsule structures in capsular types with similar *cps* gene content.

K14 and K64 showed similarity in their capsule structures, and cross-reacting between the two types has been reported as well. Similarity between the two *cps* were found: non-initial GTs (WcoU and WcoT), pyruvyl transferase (WcoV), Wzx, Wzy and a hypothetical protein. We suggest that WcoU, WcoT and WcoV are responsible for the synthesis of the identical side chains in these two types. In terms of the distinct backbone of the two types, each should contain three GTs for the sugar linkages; however, only two GTs (WcsP and WcsQ) were found in K14 and two in K64 (WcsF and WbaZ).


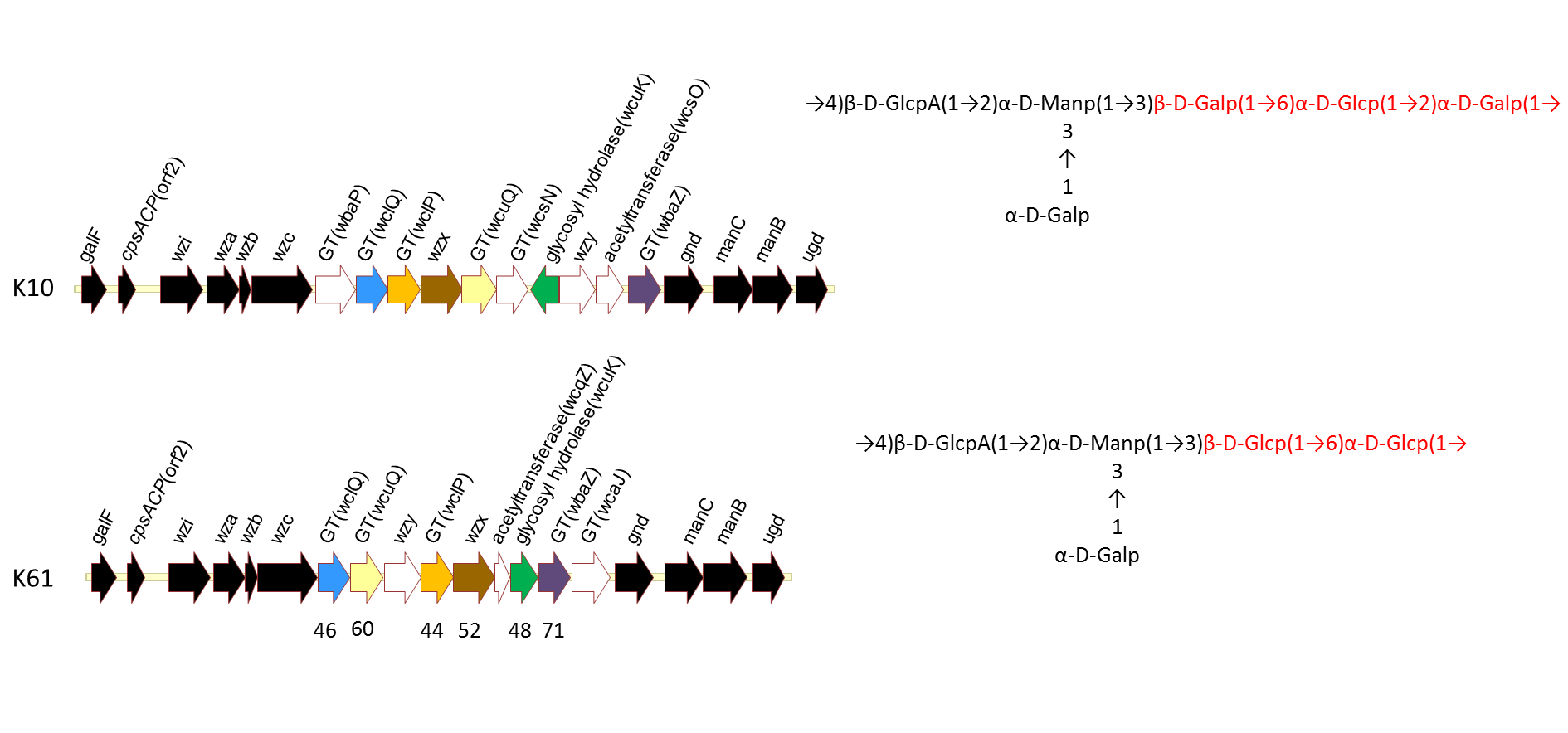


**Supplementary Figure S1b**. Comparison of *cps* gene clusters and capsule structures in capsular types with similar *cps* gene content.

Serological cross reactions between K10 and K61 were reported previously, and their capsule structures exhibit similarity. Analyzing the genetic composition of the *cps* loci in the two types indicated that four non-initial GTs (WbaZ, WcuQ, WclP and WclQ), Wzx and the glycosyl hydrolase wcuK of K10 showed similarity with products from K61. Only one GT (WcsN) was exclusively present in K10, but more than one linkage differed from those of K61. Whether GTs clustered into the same HGs are responsible for distinct linkages formation, remained to be determined.


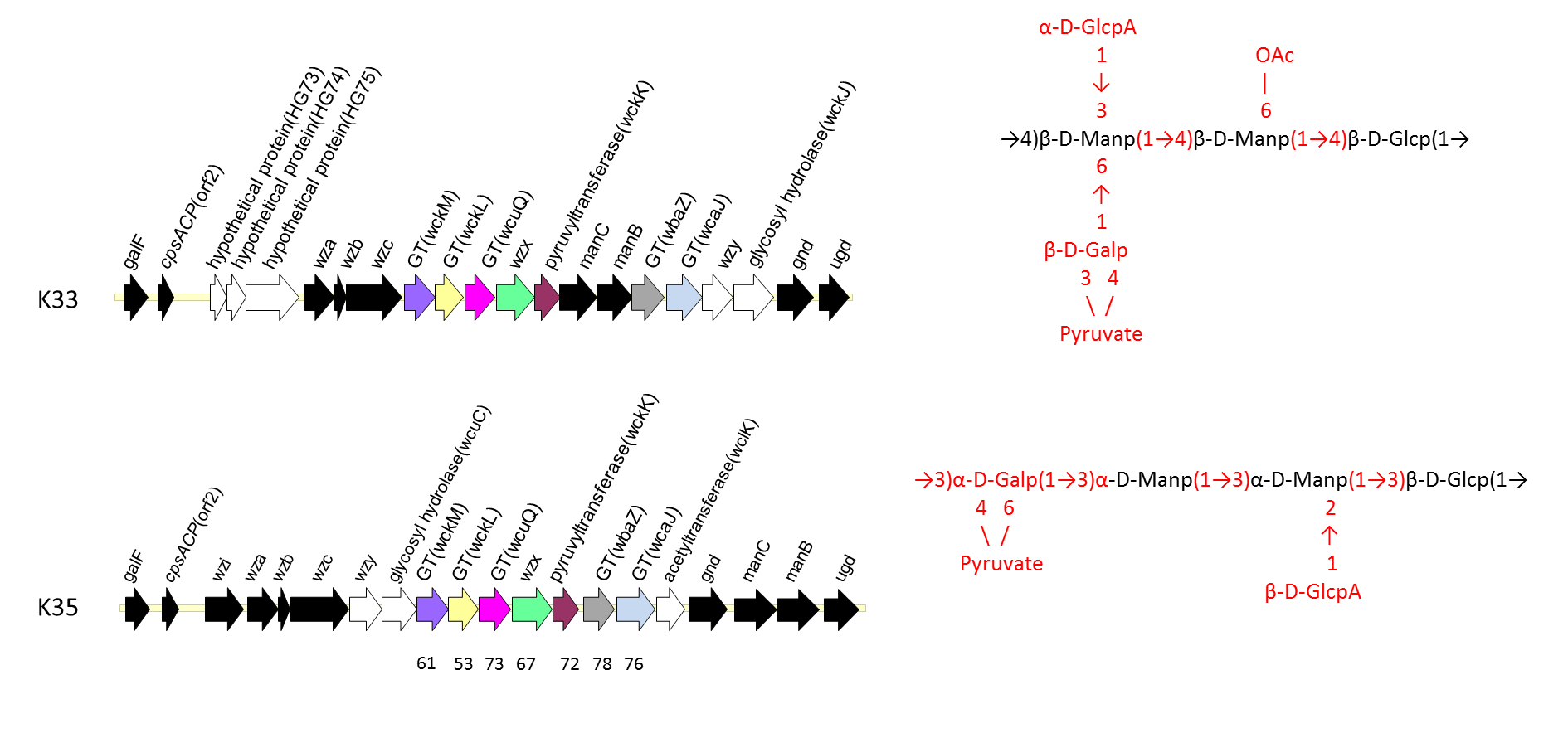


**Supplementary Figure S1c**. Comparison of *cps* gene clusters and capsule structures in capsular types with similar *cps* gene content.

K33 and K35 show high similarity in their *cps* regions (four non-initial GTs, one pyruvyl transferase and the Wzx of K33 exhibited 53%-78% amino acid identity to the corresponding proteins in K35). However, based on the previously documented chemical structures of these capsules, there are no identical sugar linkages in the two capsules, despite cross-reaction occurring between the two types.

**
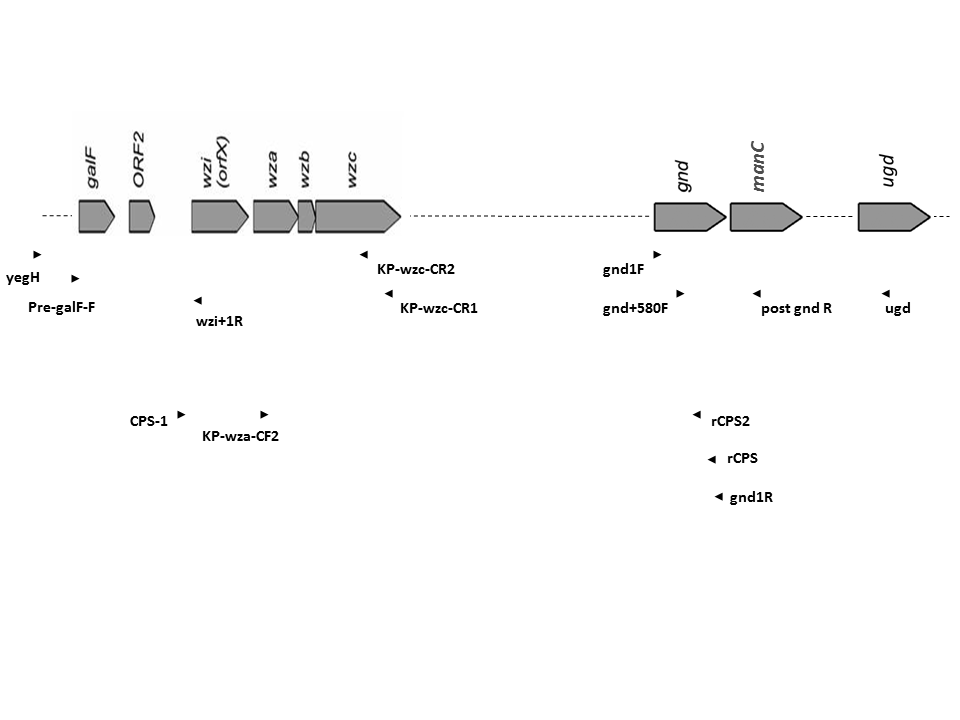
**

**Supplementary Figure S2.** Locations of primers used for *cps* region PCR amplification.

Primers are indicated by arrowheads.

**Supplementary Table S1**. ***Klebsiella* spp. 79 capsular types for *cps* analysis**

| Capsular type | Strain | Accession no. | Species | Method to define K-type |
| --- | --- | --- | --- | --- |
| K1 | NTUH-K2044^a^ | AB924547 | *K. pneumoniae* | Serotyping and *cps* genotyping[^11^](#_ENREF_11) |
| K2 | VGH525^a^ | AB371296 | *K. pneumoniae* | Serotyping |
| K3 | SB3432^a^ | FQ311478 | *K. pneumoniae* | *cps* genotyping[^14^](#_ENREF_14) |
| K4 | D5050^b, d^ | AB924548 | *K. pneumoniae* subsp. *ozaenae* | - |
| K5 | VGH404^a^ | AB371292 | *K. pneumoniae* subsp. *ozaenae* | Serotyping |
| K6 | F052^b, d^ | AB924549 | *K. pneumoniae* subsp. *ozaenae* | - |
| K7 | Aerogenes 4140^b, d^ | AB924550 | *K. pneumoniae* | - |
| K8 | Klebsiella 1015^b, d^ | AB924551 | *K. pneumoniae* | - |
| K9 | VGH484^a^ | AB371293 | *K. pneumoniae* | Serotyping |
| K10 | Klebsiella 919^b, d^ | AB924552 | *K. pneumoniae* | - |
| K11 | Klebsiella 390^b, d^ | AB924553 | *K. pneumoniae* | - |
| K12 | Klebsiella 313^b, d^ | AB924554 | *K. pneumoniae* | - |
| K13 | BIDMC 47 | AB924555/JCMR01000008^e^ | *K. pneumoniae* | *cps* genotyping |
| K14 | VGH916^a^ | AB371294 | *K. pneumoniae* | Serotyping |
| K15 | Mich. 61^c, d^ | AB924556 | *K. pneumoniae* | - |
| K16 | 2069/49^a, d^ | AB742228 | *K. pneumoniae* | *-* |
| K17 | 2005/49^b, d^ | AB924557 | *K. pneumoniae* | - |
| K18 | 1754/49^b, d^ | AB924558 | *K. pneumoniae* | - |
| K19 | 293/50^b, d^ | AB924559 | *K. pneumoniae* | - |
| K20 | NK8^a^ | AB371289 | *K. pneumoniae* | Serotyping |
| K21 | 1702/49^b, d^ | AB924560 | *K. pneumoniae* | - |
| K22 | 1996/49^a, d^ | AB819893 | *K. pneumoniae* | *-* |
| K23 | 2812/50^c, d^ | AB924561 | *K. pneumoniae* | - |
| K24 | 1680/49^b, d^ | AB924562 | *K. pneumoniae* | - |
| K25 | 2002/49^b, d^ | AB924563 | *K. pneumoniae* | - |
| K26 | 5884^b, d^ | AB924564 | *K. oxytoca* | - |
| K27 | 6613^b, d^ | AB924565 | *K. pneumoniae* | - |
| K28 | 5758^b, d^ | AB924566 | *K. pneumoniae* | - |
| K29 | 5725y^b, d^ | AB924567 | *K. oxytoca* | - |
| K30 | 7824^b, d^ | AB924568 | *K. pneumoniae* | - |
| K31 | 6258^b, d^ | AB924569 | *K. pneumoniae* | - |
| K32 | 6837^b, d^ | AB924570 | *K.* (*Raoultella*) *ornithinolytica* | - |
| K33 | 6168^b, d^ | AB924571 | *K. pneumoniae* | - |
| K34 | 7522^b, d^ | AB924572 | *K. pneumoniae* | - |
| K35 | 7444^b, d^ | AB924573 | *K.* (*Raoultella*) *planticola* | - |
| K36 | 8306^b, d^ | AB924574 | *K. pneumoniae* | - |
| K37 | 8238^c, d^ | AB924575 | *K. pneumoniae* | - |
| K38 | 8414^b, d^ | AB924576 | *K. pneumoniae* | - |
| K39 | 7749^a, d^ | AB742230 | *K. pneumoniae* | *-* |
| K40 | 8588^b, d^ | AB924577 | *K. pneumoniae* | - |
| K41 | 6177^b, d^ | AB924578 | *K. michiganensis* | - |
| K42 | 1702^b, d^ | AB924579 | *K. pneumoniae* | - |
| K43 | 2482^b, d^ | AB924580 | *K. pneumoniae* | - |
| K44 | 7730^b, d^ | AB924581 | *K.* (*Raoultella*) *ornithinolytica* | - |
| K45 | 8464^c, d^ | AB924582 | *K. pneumoniae* | - |
| K46 | 5281^b, d^ | AB924583 | *K. pneumoniae* | - |
| K47 | 9682^b, d^ | AB924584 | *K. pneumoniae* | - |
| K48 | 1196^b, d^ | AB924585 | *K. variicola* | - |
| K49 | 6115^b, d^ | AB924586 | *K. variicola* | - |
| K50 | 1303/50^c, d^ | AB924587 | *K. pneumoniae* II-B | - |
| K51 | 4715/50^b, d^ | AB924588 | *K. pneumoniae* | - |
| K52 | MGH78578^a^ | AB924589 | *K. pneumoniae* | Serotyping[^11^](#_ENREF_11) |
| K53 | 1756/51^b, d^ | AB924590 | *K. variicola* | - |
| K54 | NTUH-KP35^c^ | AB924591 | *K. variicola* | *cps* genotyping[^15^](#_ENREF_15) |
| K55 | 3985/51^b, d^ | AB924592 | *K. pneumoniae* | - |
| K56 | 3534/51^b, d^ | AB924593 | *K. variicola* | - |
| K57 | A1142^c^ | AB924594 | *K. variicola* | *cps* genotyping[^16^](#_ENREF_16) |
| K58 | 636/52^b, d^ | AB924595 | *K. variicola* | - |
| K59 | 2212/52^b, d^ | AB924596 | *K. michiganensis* | - |
| K60 | 4463/52^b, d^ | AB924597 | *K. pneumoniae* II-B | - |
| K61 | 5710/52^b, d^ | AB924598 | *K. pneumoniae* | - |
| K62 | VGH698^a^ | AB371295 | *K. pneumoniae* | Serotyping |
| K63 | 5845/52^b, d^ | AB924599 | *K. pneumoniae* | - |
| K64 | NCTC 8172^b, d^ | AB924600 | *K. pneumoniae* | - |
| K65 | SW4^b, d^ | AB924601 | *K.* (*Raoultella*) *terrigena* | - |
| K66 | 438(3a)^b, d^ | AB924602 | *K. michiganensis* | - |
| K67 | 264(1)^b, d^ | AB924603 | *K.* (*Raoultella*) *terrigena* | - |
| K68 | 265(1)^b, d^ | AB924604 | *K.* (*Raoultella*) *terrigena* | - |
| K69 | 889^b, d^ | AB924605 | *K.* (*Raoultella*) *terrigena* | - |
| K70 | 167^b, d^ | AB924606 | *K. michiganensis* | - |
| K71 | 4349^b, d^ | AB924607 | *K. variicola* | - |
| K72 | 1205^b, d^ | AB924608 | *K.* (*Raoultella*) *ornithinolytica* | - |
| K74 | 371^b, d^ | AB924609 | *K. oxytoca* | - |
| K79 | 325^c, d^ | AB924610 | *K.* (*Raoultella*) *planticola* | - |
| K80 | 708^b, d^ | AB924611 | *K. pneumoniae* II-B | - |
| K81 | 370^b, d^ | AB924612 | *K. pneumoniae* | - |
| K82 | 3454-70^b, d^ | AB924613 | *K. pneumoniae* | - |
| KN1 | A1517^c^ | AB924614 | *K. pneumoniae* | - |
| KN2 | NK29^a^ | AB371290 | *K. pneumoniae* | Serotyping |

Note: a, complete *cps* (capsular polysaccharide synthesis) sequences (from *galF* to *ugd*) were obtained from Genbank; b, *cps* gene clusters were resolved in this study; c, 3’*cps* sequences were extended in this study; d, reference strain; e, *cps* sequences of BIDMC 47 is under accession no. AB924555 and genome sequences of BIDMC 47 is under JCMR01000008.

**Supplementary Table S2**. **Homology groups (HGs) and the members**

| HG | No. of members | Predicted product | Gene | Capsular types | Note |
| --- | --- | --- | --- | --- | --- |
| 1 | 81 | capsule polysaccharide export protein | wza | All types | including 2 truncated wza of K4^a^ |
| 2 | 79 | UDP-glucose pyrophosphorylase | galF | All types |  |
| 3 | 79 | gluconate-6-phosphate dehydrogenase | gnd | All types except K50 | Including 2 gnd of K41 |
| 4 | 78 | outer membrane protein(surface assembly of capsule) | wzi | All types except K33 and K40 | including 2 truncated wzi of K39^b^ |
| 5 | 78 | tyrosine-protein kinase | wzc | All types except K15 and K50 | Including 2 wzc of K4 |
| 6 | 77 | acid phosphatase | cpsACP | All types except K4 and K65 | including a truncated cpsACP of K81 |
| 7 | 78 | protein tyrosine phosphatase | wzb | All types except K15 and K50 | including 2 wzb of K4 |
| 8 | 44 | mannose-1-phosphate guanylyltransferase | manC | 1,2,3,4,5,6,7,10,13,14,16,20,21,24,26,28,29,30,31,33,35,39,40,42,43,46,49,53,54,57,58,59,60,61,62,63,64,65,66,67,68,69,74,80 |  |
| 9 | 43 | phosphomannomutase | manB | 1,2,3,5,6,7,10,13,14,16,20,21,24,  26,28,29,30,31,33,35,39,40,42,43,46,49,53,54,57,58,59,60,61,62,63,64,65,66,67,68,69,74,80 |  |
| 10 | 40 | undecaprenolphosphate hexose-1-P transferase | wcaJ | 1,2,4,5,6,7,8,11,13,14,16,17,22,23,24,25,28,30,31,33,34,35,37,39,44,45,48,54,55,58,59,60,61,64,65,67,69,71,72,82 |  |
| 11 | 39 | undecaprenolphosphate hexose-1-P transferase | wbaP | 3,9,10,12,15,18,19,20,21,26,27,29,32,36,38,40,41,42,43,46,47,49,50,51,52,53,56,57,62,63,66,68,70,74,79,80,81,N1,N2 |  |
| 12 | 30 | glucose-1-phosphate thymidylyl-transferase | rmlA | 9,12,14,17,18,19,23,32,34,36,40,  41,44,45,47,48,52,53,55,56,64,65,67,70,71,72,80,81,N1 |  |
| 13 | 30 | dTDP-6-deoxy-L-mannose dehydrogenase | rmlD | 9,12,14,17,18,19,23,32,34,36,40,  41,44,45,47,48,52,53,55,56,64,65,67,70,71,72,80,81,N1 |  |
| 14 | 30 | dTDP-6-deoxy-D-glucose-3,5-epimerase | rmlC | 9,12,14,17,18,19,23,32,34,36,40,  41,44,45,47,48,52,53,55,56,64,65,67,70,71,72,80,81,N1 |  |
| 15 | 29 | dTDP-D-glucose-4,6-dehydratase | rmlB | 9,12,14,17,18,19,23,32,34,36,40,  41,44,45,47,48,52,53,55,56,64,67,70,71,72,80,81,N1 |  |
| 16 | 29 | glycosyl transferase | wbaZ | 3,7,10,20,21,24,26,28,29,31,33,35,39,40,43,50,53,57,59,60,61,62,64,65,66,67,68,74,80 |  |
| 17 | 19 | glycosyl transferase | wcaA | 9, 12, 18, 23, 32, 34, 36, 41, 44, 45, 47, 48, 52, 55, 71, 72, 79, 81, N1 |  |
| 18 | 16 | glycosyl hydrolase | wcuC | 3, 7, 11, 21, 24, 26, 28, 31, 35, 39, 46, 56, 65, 67, 68, 70 |  |
| 19 | 14 | glycosyl transferase | wcuD | 2, 4, 13, 21, 24, 26, 29, 30, 40, 46, 66, 69, 74, 80 |  |
| 20 | 14 | glycosyl transferase | wcuE | 3, 7, 21, 24, 26, 28, 29, 39, 40, 43, 53, 65, 74, 80 |  |
| 21 | 13 | flippase | wzx | 8, 9, 15, 20, 23, 28, 38, 45, 51, 55, 59, 71, 82 |  |
| 22 | 12 | flippase | wzx | 18, 19, 24, 39, 41, 43, 44, 47, 52, 53, 60, N2 |  |
| 23 | 10 | glycosyl transferase | wcuF | 2, 4, 11, 13, 19, 46, 49, 51, 70, 82 |  |
| 24 | 8 | flippase | wzx | 14, 31, 33, 35, 36, 56, 64, 66 |  |
| 25 | 7 | acetyltransferase | wcuG | 4, 22, 24, 30, 37, 37, 69 |  |
| 26 | 7 | glycosyl transferase | wcuH | 18, 26, 44, 50, 60, 79, N2 |  |
| 27 | 6 | flippase | wzx | 22, 37, 65, 70, 80, N1 |  |
| 28 | 6 | glycosyl transferase | wcuI | 4, 46, 49, 59, 66, 67 |  |
| 29 | 6 | transposase | tnp | 4,40,41 | including 4 transposases of K4 |
| 30 | 6 | glycosyl transferase | wcaI | 1, 6, 16, 54, 58, 63 |  |
| 31 | 6 | fucose synthase | wcaG | 1, 6, 16, 54, 58, 63 |  |
| 32 | 6 | GDP-D-mannose 4, 6-dehydratase | gmd | 1, 6, 16, 54, 58, 63 |  |
| 33 | 5 | pyruvyl transferase | wcuJ | 7, 27, 31, 36, 56 |  |
| 34 | 5 | glycosyl hydrolase | wcuK | 10, 53, 61, 62, 64 |  |
| 35 | 5 | UDP-galactopyranose mutase | glf | 12, 14, 29, 41, 42 |  |
| 36 | 5 | pyruvyl transferase | wcuL | 13, 26, 30, 69, 74 |  |
| 37 | 5 | glycosyl transferase | wcuM | 18, 36, 44, 79, 81 |  |
| 38 | 5 | transposase | tnp | 40,57,69 | including 3 transposases of K40 |
| 39 | 4 | pyruvyl transferase | wcuN | 65, 70, 80, N1 |  |
| 40 | 4 | glycosyl transferase | wcuO | 39, 42, 43, 65 |  |
| 41 | 4 | glycosyl transferase | wcuP | 31, 45, 71, N1 |  |
| 42 | 4 | glycosyl transferase | wcuQ | 33, 35, 10, 61 |  |
| 43 | 4 | flippase | wzx | 3, 7, 27, 46 |  |
| 44 | 4 | flippase | wzx | 2, 13, 74, 79 |  |
| 45 | 3 | glycosyl transferase | wcuR | 72, 79, N1 |  |
| 46 | 3 | capsular repeat unit polymerase | wzy | 52, 53, 81 |  |
| 47 | 3 | glycosyl transferase | wcuS | 52, 65, 80 |  |
| 48 | 3 | glycosyl transferase | wcuT | 47, 53, 70 |  |
| 49 | 3 | capsular repeat unit polymerase | wzy | 42, 59, 65 |  |
| 50 | 3 | capsular repeat unit polymerase | wzy | 36, 48, 67 |  |
| 51 | 3 | capsular repeat unit polymerase | wzy | 27, 38, N2 |  |
| 52 | 3 | glycosyl transferase | wcuU | 26, 50, 79 |  |
| 53 | 3 | glycosyl transferase | wcuV | 24, 43, 46 |  |
| 54 | 3 | glycosyl transferase | wcuW | 22, 25, 37 |  |
| 55 | 3 | glycosyl transferase | wckA | 22, 23, 37 |  |
| 56 | 3 | capsular repeat unit polymerase | wzy | 22, 25, 37 |  |
| 57 | 3 | GDP-mannose mannosyl hydrolase | wcaH | 1, 16, 54 |  |
| 58 | 3 | capsular repeat unit polymerase | wzy | 3, 24, 28 |  |
| 59 | 3 | acetyltransferase | wckB | 6, 16, 54 |  |
| 60 | 3 | glycosyl transferase | wckC | 7, 28, 66 |  |
| 61 | 3 | glycosyl transferase | wckD | 7, 28, 62 |  |
| 62 | 3 | glycosyl transferase | wckE | 9, 34, 36 |  |
| 63 | 3 | glycosyl transferase | wckF | 12, 18, 41 |  |
| 64 | 3 | glycosyl transferase | wckG | 12, 29, 42 |  |
| 65 | 3 | pyruvyl transferase | wckH | 12, 29, 42 |  |
| 66 | 3 | flippase | wzx | 12, 29, 42 |  |
| 67 | 4 | glycosyl transferase | wcaN | 15, 20, 38, 52 |  |
| 68 | 3 | Glycosyl hydrolase | wckI | 15, 27, N2 |  |
| 69 | 2 | Glycosyl hydrolase | wckJ | 33, 59 |  |
| 70 | 2 | pyruvyl transferase | wckK | 33, 35 |  |
| 71 | 2 | glycosyl transferase | wckL | 33, 35 |  |
| 72 | 2 | glycosyl transferase | wckM | 33, 35 |  |
| 73 | 2 | putative lipoprotein | HG73 | 33, 40 |  |
| 74 | 2 | hypothetical protein | HG74 | 33, 40 |  |
| 75 | 2 | putative lipoprotein | HG75 | 33, 40 |  |
| 76 | 2 | tail fiber | HG76 | 30, 69 |  |
| 77 | 2 | glycosyl transferase | wckN | 30, 69 |  |
| 78 | 2 | glycosyl transferase | wckO | 30, 69 |  |
| 79 | 2 | glycosyl transferase | wckP | 30, 69 |  |
| 80 | 2 | capsular repeat unit polymerase | wzy | 30, 69 |  |
| 81 | 2 | flippase | wzx | 30, 69 |  |
| 82 | 2 | glycosyl transferase | wckQ | 27, 46 |  |
| 83 | 2 | glycosyl transferase | wckR | 27, 38 |  |
| 84 | 2 | glycosyl transferase | wckS | 27, 38 |  |
| 85 | 2 | glycosyl transferase | wckT | 25, 36 |  |
| 86 | 2 | glycosyl transferase | wckU | 23, 48 |  |
| 87 | 2 | hypothetical protein | HG87 | 22, 37 |  |
| 88 | 2 | capsular repeat unit polymerase | wzy | 79, N1 |  |
| 89 | 2 | hypothetical protein | HG89 | 79, N1 |  |
| 90 | 2 | hypothetical protein | HG90 | 74, 80 |  |
| 91 | 2 | capsular repeat unit polymerase | wzy | 74, 80 |  |
| 92 | 2 | glycosyl transferase | wckV | 59, 82 |  |
| 93 | 2 | glycosyl transferase | wckW | 57, 68 |  |
| 94 | 2 | glycosyl transferase | wckX | 57, 68 |  |
| 95 | 2 | flippase | wzx | 57, 68 |  |
| 96 | 2 | glycosyl transferase | wckY | 56, 82 |  |
| 97 | 2 | glycosyl transferase | wckZ | 53, 67 |  |
| 98 | 2 | glycosyl transferase | wclH | 53, 67 |  |
| 99 | 2 | transposase | tnp | 50 | including 2 transposases of K50 |
| 100 | 2 | flippase | wzx | 48, 49 |  |
| 101 | 2 | capsular repeat unit polymerase | wzy | 45, 72 |  |
| 102 | 2 | glycosyl transferase | wclI | 45, 71 |  |
| 103 | 2 | glycosyl transferase | wclJ | 44, 81 |  |
| 104 | 2 | acetyltransferase | wclK | 35, 49 |  |
| 105 | 2 | capsular repeat unit polymerase | wzy | 35, 46 |  |
| 106 | 2 | glycosyl transferase | wclL | 34, 48 |  |
| 107 | 2 | glycosyl transferase | wclM | 12, 41 |  |
| 108 | 2 | glycosyl transferase | wclN | 12, 41 |  |
| 109 | 2 | glycosyl transferase | wclO | 11, N2 |  |
| 110 | 2 | capsular repeat unit polymerase | wzy | 11, 82 |  |
| 111 | 2 | flippase | wzx | 10, 61 |  |
| 112 | 2 | glycosyl transferase | wclP | 10, 61 |  |
| 113 | 2 | glycosyl transferase | wclQ | 10, 61 |  |
| 114 | 2 | glycosyl transferase | wclR | 9, 70 |  |
| 115 | 2 | glycosyl transferase | wclS | 7, 28 |  |
| 116 | 2 | pyruvyl transferase | wclT | 6, 46 |  |
| 117 | 2 | glycosyl transferase | wclU | 6, 54 |  |
| 118 | 2 | pyruvyl transferase | wclV | 3, 68 |  |
| 119 | 2 | glycosyl transferase | wclW | 3, 49 |  |
| 120 | 2 | hypothetical protein | HG120 | 2, 13 |  |
| 121 | 2 | capsular repeat unit polymerase | wzy | 2, 13 |  |
| 122 | 2 | glycosyl transferase | wclX | 2, 13 |  |
| 123 | 2 | pyruvyl transferase | wclY | 1, 58 |  |
| 124 | 2 | flippase | wzx | 1, 58 |  |
| 125 | 2 | capsular repeat unit polymerase | wzy | 12, 41 |  |
| 126 | 2 | pyruvyl transferase | wclZ | 22, 37 |  |
| 127 | 2 | nitroreductase | HG127 | 22, 37 |  |
| 128 | 2 | glycosyl transferase | wcmA | 22, 37 |  |
| 129 | 2 | hypothetical protein | HG129 | 12, 41 |  |
| 130 | 2 | glycosyl transferase | wcaO | 20, 60 |  |
| 131 | 2 | glycosyl transferase | wcmY | 19, 70 |  |
| 132 | 2 | hypothetical protein | HG132 | 18, 81 |  |
| 133 | 2 | flippase | wzx | 17, 81 |  |
| 134 | 2 | glycosyl transferase | wcmZ | 17, 81 |  |
| 135 | 2 | glycosyl transferase | wcoS | 16, 54 |  |
| 136 | 2 | glycosyl transferase | GT-wceM | 15, N2 |  |
| 137 | 2 | glycosyl transferase | GT-wceN | 15, N2 |  |
| 138 | 2 | glycosyl transferase | wcoT | 14, 64 |  |
| 139 | 2 | glycosyl transferase | wcoU | 14, 64 |  |
| 140 | 2 | capsular repeat unit polymerase | wzy | 14, 64 |  |
| 141 | 2 | hypothetical protein | HG141 | 14, 64 |  |
| 142 | 2 | pyruvyltransferase | wcoV | 14, 64 |  |
| 143 | 2 | glycosyl transferase | wcoW | 13, 74 |  |
| 144 | 1 | flippase | wzx | 25 |  |
| 145 | 1 | flippase | wzx | 26 |  |
| 146 | 1 | glycosyl transferase | wcoX | 52 |  |
| 147 | 1 | glycosyl transferase | wcoY | 52 |  |
| 148 | 1 | acetyltransferase | wcoZ | 51 |  |
| 149 | 1 | hypothetical protein | HG149 | 51 |  |
| 150 | 1 | glycosyl transferase | wcpA | 51 |  |
| 151 | 1 | capsular repeat unit polymerase | wzy | 51 |  |
| 152 | 1 | glycosyl transferase | wcpB | 51 |  |
| 153 | 1 | glycosyl transferase | wcpC | 50 |  |
| 154 | 1 | glycosyl transferase | wcpD | 50 |  |
| 155 | 1 | glycosyl transferase | wcpE | 50 |  |
| 156 | 1 | hypothetical protein(kinase) | HG156 | 50 |  |
| 157 | 1 | hypothetical protein | HG157 | 49 |  |
| 158 | 1 | acetyltransferase | wcpF | 49 |  |
| 159 | 1 | carbohydrate lyase | HG159 | 52 |  |
| 160 | 1 | acetyltransferase | wcpG | 52 |  |
| 161 | 1 | flippase | wzx | 54 |  |
| 162 | 1 | capsular repeat unit polymerase | wzy | 54 |  |
| 163 | 1 | glycosyl transferase | wcpH | 55 |  |
| 164 | 1 | acetyltransferase | wcpI | 55 |  |
| 165 | 1 | capsular repeat unit polymerase | wzy | 55 |  |
| 166 | 1 | glycosyl transferase | wcpJ | 55 |  |
| 167 | 1 | hypothetical protein | HG167 | 55 |  |
| 168 | 1 | glycosyl transferase | wcpK | 56 |  |
| 169 | 1 | glycosyl transferase | wcpL | 56 |  |
| 170 | 1 | glycosyl transferase | wcpM | 56 |  |
| 171 | 1 | capsular repeat unit polymerase | wzy | 56 |  |
| 172 | 1 | capsular repeat unit polymerase | wzy | 57 |  |
| 173 | 1 | acetyltransferase | wcpN | 39 |  |
| 174 | 1 | glycosyl transferase | wcpO | 40 |  |
| 175 | 1 | glycosyl transferase | wcpP | 40 |  |
| 176 | 1 | glycosyl transferase | wcpQ | 40 |  |
| 177 | 1 | capsular repeat unit polymerase | wzy | 40 |  |
| 178 | 1 | glycosyl transferase | wcpR | 40 |  |
| 179 | 1 | flippase | wzx | 40 |  |
| 180 | 1 | glycosyl transferase | wcpS | 40 |  |
| 181 | 1 | hypothetical protein | HG181 | 40 |  |
| 182 | 1 | glycosyl transferase | wcpT | 41 |  |
| 183 | 1 | glycosyl transferase | wcpU | 41 |  |
| 184 | 1 | glycosyl hydrolase | wcpV | 42 |  |
| 185 | 1 | glycosyl transferase | wcpW | 42 |  |
| 186 | 1 | glycosyl transferase | wcpX | 42 |  |
| 187 | 1 | capsular repeat unit polymerase | wzy | 43 |  |
| 188 | 1 | glycosyl hydrolase | wcpY | 43 |  |
| 189 | 1 | acetyltransferase | wcpZ | 43 |  |
| 190 | 1 | capsular repeat unit polymerase | wzy | 44 |  |
| 191 | 1 | hypothetical protein | HG191 | 44 |  |
| 192 | 1 | acetyltransferase | wcqA | 44 |  |
| 193 | 1 | glycosyl transferase | wcqB | 45 |  |
| 194 | 1 | hypothetical protein | HG194 | 47 |  |
| 195 | 1 | glycosyl transferase | wcqC | 47 |  |
| 196 | 1 | capsular repeat unit polymerase | wzy | 47 |  |
| 197 | 1 | glycosyl transferase | wcqD | 48 |  |
| 198 | 1 | hypothetical protein | HG198 | 48 |  |
| 199 | 1 | acetyltransferase | wcqE | 48 |  |
| 200 | 1 | capsular repeat unit polymerase | wzy | 49 |  |
| 201 | 1 | capsular repeat unit polymerase | wzy | 66 |  |
| 202 | 1 | hypothetical protein | HG202 | 66 |  |
| 203 | 1 | coenzyme_F420_hydrogenase | HG203 | 66 |  |
| 204 | 1 | pyruvyltransferase | wcqF | 66 |  |
| 205 | 1 | glycosyl transferase | wcqG | 67 |  |
| 206 | 1 | glycosyl transferase | wcqH | 67 |  |
| 207 | 1 | flippase | wzx | 67 |  |
| 208 | 1 | capsular repeat unit polymerase | wzy | 68 |  |
| 209 | 1 | UDP galacturonate 4- epimerase | HG209 | 68 |  |
| 210 | 1 | acetyltransferase | wcqI | 69 |  |
| 211 | 1 | capsular repeat unit polymerase | wzy | 70 |  |
| 212 | 1 | glycosyl transferase | wcqJ | 71 |  |
| 213 | 1 | hypothetical protein | HG213 | 71 |  |
| 214 | 1 | capsular repeat unit polymerase | wzy | 71 |  |
| 215 | 1 | glycosyl transferase | wcqK | 71 |  |
| 216 | 1 | hypothetical protein | HG216 | 25 |  |
| 217 | 1 | flippase | wzx | 72 |  |
| 218 | 1 | pyruvyltransferase | wcqL | 72 |  |
| 219 | 1 | hypothetical protein | HG219 | 72 |  |
| 220 | 1 | acid phosphatase | cpsACP | 81 | truncated cpsACP of K81 |
| 221 | 1 | transposase | tnp | 81 |  |
| 222 | 1 | glycosyl transferase | wcqM | 81 |  |
| 223 | 1 | acetyltransferase | wcqN | 82 |  |
| 224 | 1 | hypothetical protein | HG224 | 82 |  |
| 225 | 1 | glycosyl transferase | wcqO | 82 |  |
| 226 | 1 | glycosyl transferase | wcqP | N1 |  |
| 227 | 1 | glycosyl transferase | wcqQ | N2 |  |
| 228 | 1 | hypothetical protein | HG228 | 57 |  |
| 229 | 1 | acetyltransferase | wcqR | 57 |  |
| 230 | 1 | capsular repeat unit polymerase | wzy | 58 |  |
| 231 | 1 | glycosyl transferase | wcqS | 58 |  |
| 232 | 1 | glycosyl transferase | wcqT | 58 |  |
| 233 | 1 | hypothetical protein | HG233 | 58 |  |
| 234 | 1 | acetyltransferase | wcqU | 58 |  |
| 235 | 1 | glycosyl transferase | wcqV | 59 |  |
| 236 | 1 | capsular repeat unit polymerase | wzy | 60 |  |
| 237 | 1 | glycosyl transferase | wcqW | 60 |  |
| 238 | 1 | glycosyl transferase | wcqX | 60 |  |
| 239 | 1 | glycosyl transferase | wcqY | 60 |  |
| 240 | 1 | hypothetical protein | HG240 | 60 |  |
| 241 | 1 | capsular repeat unit polymerase | wzy | 61 |  |
| 242 | 1 | acetyltransferase | wcqZ | 61 |  |
| 243 | 1 | glycosyl transferase | wcsA | 62 |  |
| 244 | 1 | capsular repeat unit polymerase | wzy | 62 |  |
| 245 | 1 | acetyltransferase | wcsB | 62 |  |
| 246 | 1 | glycosyl transferase | wcsC | 62 |  |
| 247 | 1 | flippase | wzx | 62 |  |
| 248 | 1 | acetyltransferase | wcsD | 63 |  |
| 249 | 1 | flippase | wzx | 63 |  |
| 250 | 1 | capsular repeat unit polymerase | wzy | 63 |  |
| 251 | 1 | glycosyl transferase | wcsE | 63 |  |
| 252 | 1 | hypothetical protein | HG252 | 63 |  |
| 253 | 1 | hypothetical protein | HG253 | 63 |  |
| 254 | 1 | glycosyl transferase | wcsF | 64 |  |
| 255 | 1 | acid phosphatase | cpsACP | 65 |  |
| 256 | 1 | GDP-mannose mannosyl hydrolase | NudD | 6 |  |
| 257 | 1 | flippase | wzx | 6 |  |
| 258 | 1 | hypothetical protein | HG258 | 6 |  |
| 259 | 1 | capsular repeat unit polymerase | wzy | 7 |  |
| 260 | 1 | capsular repeat unit polymerase | wzy | 8 |  |
| 261 | 1 | acetyltransferase | wcsG | 8 |  |
| 262 | 1 | glycosyl transferase | wcsH | 8 |  |
| 263 | 1 | pyruvyltransferase | wcsI | 8 |  |
| 264 | 1 | glycosyl transferase | wcsJ | 8 |  |
| 265 | 1 | glycosyl transferase | wcsK | 8 |  |
| 266 | 1 | glycosyl transferase | wcsL | 8 |  |
| 267 | 1 | glycosyl transferase | wcsM | 8 |  |
| 268 | 1 | capsular repeat unit polymerase | wzy | 9 |  |
| 269 | 1 | hypothetical protein | HG269 | 9 |  |
| 270 | 1 | glycosyl transferase | wcsN | 10 |  |
| 271 | 1 | capsular repeat unit polymerase | wzy | 10 |  |
| 272 | 1 | acetyltransferase | wcsO | 10 |  |
| 273 | 1 | hypothetical protein | HG273 | 14 |  |
| 274 | 1 | glycosyl transferase | wcsP | 14 |  |
| 275 | 1 | glycosyl transferase | wcsQ | 14 |  |
| 276 | 1 | transposase | tnp | 15 |  |
| 277 | 1 | glycosyl transferase | wcsR | 15 |  |
| 278 | 1 | capsular repeat unit polymerase | wzy | 15 |  |
| 279 | 1 | transposase | tnp | 15 |  |
| 280 | 1 | transposase | tnp | 15 |  |
| 281 | 1 | flippase | wzx | 16 |  |
| 282 | 1 | capsular repeat unit polymerase | wzy | 16 |  |
| 283 | 1 | capsular repeat unit polymerase | wzy | 1 |  |
| 284 | 1 | glycosyl transferase | wcsS | 1 |  |
| 285 | 1 | acetyltransferase | wcsT | 1 |  |
| 286 | 1 | acetyltransferase | wcsU | 2 |  |
| 287 | 1 | glycosyl transferase | wcsV | 3 |  |
| 288 | 1 | transposase | tnp | 3 |  |
| 289 | 1 | acid phosphatase | cpsACP | 4 |  |
| 290 | 1 | potassium/proton_antiporter | HG290 | 4 |  |
| 291 | 1 | potassium/proton_antiporter | HG291 | 4 |  |
| 292 | 1 | CMP-N-acetylneuraminic acid synthetase | HG292 | 4 |  |
| 293 | 1 | acetylneuraminic acid synthetase | HG293 | 4 |  |
| 294 | 1 | flippase | wzx | 4 |  |
| 295 | 1 | acetyltransferase | wcsW | 4 |  |
| 296 | 1 | hypothetical protein | HG296 | 4 |  |
| 297 | 1 | capsular repeat unit polymerase | wzy | 4 |  |
| 298 | 1 | capsular repeat unit polymerase | wzy | 5 |  |
| 299 | 1 | flippase | wzx | 5 |  |
| 300 | 1 | pyruvyl transferase | wcsX | 5 |  |
| 301 | 1 | hypothetical protein | HG301 | 5 |  |
| 302 | 1 | acetyltransferase | wcsY | 5 |  |
| 303 | 1 | glycosyl transferase | wcsZ | 5 |  |
| 304 | 1 | glycosyl transferase | wctA | 5 |  |
| 305 | 1 | glycosyl transferase | wctB | 5 |  |
| 306 | 1 | transposase | tnp | 6 |  |
| 307 | 1 | transposase | tnp | 6 |  |
| 308 | 1 | glycosyl transferase | wcuB | 25 |  |
| 309 | 1 | capsular repeat unit polymerase | wzy | 6 |  |
| 310 | 1 | glycosyl transferase | wctC | 6 |  |
| 311 | 1 | glycosyl transferase | wctD | 26 |  |
| 312 | 1 | capsular repeat unit polymerase | wzy | 26 |  |
| 313 | 1 | glycosyl transferase | wctE | 27 |  |
| 314 | 1 | glycosyl transferase | wctF | 27 |  |
| 315 | 1 | glycosyl transferase | wctG | 31 |  |
| 316 | 1 | capsular repeat unit polymerase | wzy | 31 |  |
| 317 | 1 | glycosyl transferase | wctH | 31 |  |
| 318 | 1 | glycosyl transferase | wctI | 31 |  |
| 319 | 1 | flippase | wzx | 32 |  |
| 320 | 1 | pyruvyl transferase | wctJ | 32 |  |
| 321 | 1 | capsular repeat unit polymerase | wzy | 32 |  |
| 322 | 1 | glycosyl transferase | wctK | 32 |  |
| 323 | 1 | glycosyl transferase | wctL | 32 |  |
| 324 | 1 | hypothetical protein | HG324 | 32 |  |
| 325 | 1 | capsular repeat unit polymerase | wzy | 33 |  |
| 326 | 1 | capsular repeat unit polymerase | wzy | 34 |  |
| 327 | 1 | hypothetical protein | HG327 | 34 |  |
| 328 | 1 | tail fiber | HG328 | 34 |  |
| 329 | 1 | tail fiber | HG329 | 34 |  |
| 330 | 1 | tail fiber | HG330 | 36 |  |
| 331 | 1 | transposase | tnp | 38 |  |
| 332 | 1 | glycosyl transferase | wctM | 38 |  |
| 333 | 1 | hypothetical protein | HG333 | 38 |  |
| 334 | 1 | hypothetical protein | HG334 | 38 |  |
| 335 | 1 | capsular repeat unit polymerase | wzy | 39 |  |
| 336 | 1 | glycosyl transferase | wctN | 39 |  |
| 337 | 1 | glycosyl transferase | wctO | 39 |  |
| 338 | 1 | glycosyl transferase | wctP | 16 |  |
| 339 | 1 | acetyltransferase | wctQ | 16 |  |
| 340 | 1 | hypothetical protein | HG340 | 16 |  |
| 341 | 1 | capsular repeat unit polymerase | wzy | 17 |  |
| 342 | 1 | glycosyl transferase | wctR | 17 |  |
| 343 | 1 | glycosyl transferase | wctS | 17 |  |
| 344 | 1 | glycosyl transferase | wctT | 17 |  |
| 345 | 1 | glycosyl transferase | wctU | 17 |  |
| 346 | 1 | glycosyl transferase | wctV | 18 |  |
| 347 | 1 | capsular repeat unit polymerase | wzy | 18 |  |
| 348 | 1 | hypothetical protein | HG348 | 23 |  |
| 349 | 1 | glycosyl transferase | wctW | 19 |  |
| 350 | 1 | glycosyl transferase | wctX | 19 |  |
| 351 | 1 | capsular repeat unit polymerase | wzy | 19 |  |
| 352 | 1 | tail_fiber | HG352 | 19 |  |
| 353 | 1 | capsular repeat unit polymerase | wzy | 20 |  |
| 354 | 1 | acetyltransferase | wctY | 20 |  |
| 355 | 1 | glycosyl hydrolase | wctZ | 20 |  |
| 356 | 1 | capsular repeat unit polymerase | wzy | 21 |  |
| 357 | 1 | pyruvyl transferase | wcuA | 21 |  |
| 358 | 1 | glycosyl transferase | wcaL | 21 |  |
| 359 | 1 | flippase | wzx | 21 |  |
| 360 | 1 | capsular repeat unit polymerase | wzy | 23 |  |
| 361 | 1 | flippase | wzx | 50 |  |

Note: a, there are 2 truncated and 1 intact *wza* in K4; b, there are 2 *wzi* in K39 and both are truncated.

**Supplementary Table S3. Sugar composition and related genes of 79 capsular types**

Note: K-types with no available information on their CPS structures were marked in grey. The presence of gene or sugar was indicated by “+”, whereas the absence of gene or sugar was indicated by “-”. a, no *manB*; b, no *rmlB*; c, truncated gene; N/A, not available

|  | Mannose | *manCB* | Rhamnose | *rmlBADC* | Fucose | *gmd* | *wcaG* | Galacto-  furanose | *glf* | acetyl-  group | acetyl-  transferase | pyruvyl-group | pyruvyl-  transferase | Galactose | *wbaP* | Glucose | *wcaJ* | CPS structure reference |
| --- | --- | --- | --- | --- | --- | --- | --- | --- | --- | --- | --- | --- | --- | --- | --- | --- | --- | --- |
| K1 | - | + | - | - | + | + | + | - | - | + | + | + | + | - | - | + | + | [^17^](#_ENREF_17) |
| K2 | + | + | - | - | - | - | - | - | - | - | + | - | - | - | - | + | + | [^18^](#_ENREF_18) |
| K3 | + | + | - | - | - | - | - | - | - | - | - | + | + | + | + | - | - | [^19^](#_ENREF_19) |
| K4 | + | +^a^ | - | - | - | - | - | - | - | - | + | - | - | - | - | + | + | [^20^](#_ENREF_20) |
| K5 | + | + | - | - | - | - | - | - | - | + | + | + | + | - | - | + | + | [^21^](#_ENREF_21) |
| K6 | + | + | - | - | + | + | + | - | - | - | + | + | + | - | - | + | + | [^18^](#_ENREF_18) |
| K7 | + | + | - | - | - | - | - | - | - | - | - | + | + | + | - | + | + | [^22^](#_ENREF_22) |
| K8 | - | - | - | - | - | - | - | - | - | - | + | - | + | + | - | + | + | [^23^](#_ENREF_23) |
| K9 | - | - | + | + | - | - | - | - | - | - | - | - | - | + | + | - | - | [^24^](#_ENREF_24) |
| K10 | + | + | - | - | - | - | - | - | - | - | + | - | - | + | + | + | - | [^6^](#_ENREF_6) |
| K11 | - | - | - | - | - | - | - | - | - | - | - | + | - | + | - | + | + | [^25^](#_ENREF_25) |
| K12 | - | - | + | + | - | - | - | + | + | - | - | + | + | + | + | + | - | [^26^](#_ENREF_26) |
| K13 | + | + | - | - | - | - | - | - | - | - | - | + | + | + | - | + | + | [^18^](#_ENREF_18) |
| K14 | + | + | + | + | - | - | - | + | + | - | - | + | + | - | - | + | + | [^1^](#_ENREF_1) |
| K15 | - | - | - | - | - | - | - | - | - | - | - | - | - | + | + | + | - | [^27^](#_ENREF_27) |
| K16 | - | + | - | - | + | + | + | - | - | - | + | - | - | + | - | + | + | [^28^](#_ENREF_28) |
| K17 | - | - | + | + | - | - | - | - | - | - | - | - | - | - | - | + | + | [^29^](#_ENREF_29) |
| K18 | - | - | + | + | - | - | - | - | - | - | - | - | - | + | + | + | - | [^30^](#_ENREF_30) |
| K19 | - | - | + | + | - | - | - | - | - | - | - | - | - | + | + | + | - | [^31^](#_ENREF_31) |
| K20 | + | + | - | - | - | - | - | - | - | + | + | - | - | + | + | - | - | [^32^](#_ENREF_32) |
| K21 | + | + | - | - | - | - | - | - | - | - | - | + | + | + | + | - | - | [^33^](#_ENREF_33) |
| K22 | - | - | - | - | - | - | - | - | - | + | + | - | + | + | - | + | + | [^18^](#_ENREF_18) |
| K23 | - | - | + | + | - | - | - | - | - | - | - | - | - | - | - | + | + | [^34^](#_ENREF_34) |
| K24 | + | + | - | - | - | - | - | - | - | - | + | - | - | - | - | + | + | [^35^](#_ENREF_35) |
| K25 | - | - | - | - | - | - | - | - | - | - | - | - | - | + | - | + | + | [^36^](#_ENREF_36) |
| K26 | + | + | - | - | - | - | - | - | - | - | - | + | + | + | + | + | - | [^37^](#_ENREF_37) |
| K27 | - | - | - | - | - | - | - | - | - | - | - | + | + | + | + | + | - | [^38^](#_ENREF_38) |
| K28 | + | + | - | - | - | - | - | - | - | - | - | - | - | + | - | + | + | [^39^](#_ENREF_39) |
| K29 | N/A | + | N/A | - | N/A | - | - | N/A | - | N/A | - | N/A | + | N/A | + | N/A | - |  |
| K30 | + | + | - | - | - | - | - | - | - | + | + | + | + | + | - | + | + | [^40^](#_ENREF_40) |
| K31 | + | + | - | - | - | - | - | - | - | - | - | + | + | + | - | + | + | [^41^](#_ENREF_41) |
| K32 | - | - | + | + | - | - | - | - | - | - | - | + | + | + | + | - | - | [^42^](#_ENREF_42) |
| K33 | + | + | - | - | - | - | - | - | - | + | - | + | + | + | - | + | + | [^8^](#_ENREF_8) |
| K34 | - | - | + | + | - | - | - | - | - | - | - | - | - | - | - | + | + | [^43^](#_ENREF_43) |
| K35 | + | + | - | - | - | - | - | - | - | - | + | + | + | + | - | + | + | [^9^](#_ENREF_9) |
| K36 | - | - | + | + | - | - | - | - | - | - | - | + | + | + | + | + | - | [^44^](#_ENREF_44) |
| K37 | - | - | - | - | - | - | - | - | - | - | +^c^ | - | + | + | - | + | + | [^18^](#_ENREF_18) |
| K38 | - | - | - | - | - | - | - | - | - | - | - | - | - | + | + | + | - | [^45^](#_ENREF_45) |
| K39 | + | + | - | - | - | - | - | - | - | - | + | - | - | - | - | + | + | [^46^](#_ENREF_46) |
| K40 | + | + | + | + | - | - | - | - | - | - | - | - | - | + | + | - | - | [^47^](#_ENREF_47) |
| K41 | - | - | + | + | - | - | - | + | + | - | - | - | - | + | + | + | - | [^48^](#_ENREF_48) |
| K42 | N/A | + | N/A | - | N/A | - | - | N/A | - | N/A | - | N/A | + | N/A | + | N/A | - |  |
| K43 | + | + | - | - | - | - | - | - | - | - | + | - | - | + | + | - | - | [^49^](#_ENREF_49) |
| K44 | - | - | + | + | - | - | - | - | - | - | + | - | - | - | - | + | + | [^50^](#_ENREF_50) |
| K45 | - | - | + | + | - | - | - | - | - | - | - | - | - | - | - | + | + | [^51^](#_ENREF_51) |
| K46 | + | + | - | - | - | - | - | - | - | - | - | + | + | + | + | + | - | [^52^](#_ENREF_52) |
| K47 | - | - | + | + | - | - | - | - | - | - | - | - | - | + | + | - | - | [^53^](#_ENREF_53) |
| K48 | - | - | + | + | - | - | - | - | - | - | + | - | - | - | - | + | + | [^54^](#_ENREF_54) |
| K49 | + | + | - | - | - | - | - | - | - | + | + | - | - | + | + | - | - | [^55^](#_ENREF_55) |
| K50 | + | - | - | - | - | - | - | - | - | - | - | - | - | + | + | + | - | [^56^](#_ENREF_56) |
| K51 | - | - | - | - | - | - | - | - | - | - | + | - | - | + | + | + | - | [^57^](#_ENREF_57) |
| K52 | - | - | + | + | - | - | - | - | - | - | + | - | - | + | + | - | - | [^58^](#_ENREF_58) |
| K53 | + | + | + | + | - | - | - | - | - | - | - | - | - | + | + | - | - | [^59^](#_ENREF_59) |
| K54 | - | + | - | - | + | + | + | - | - | + | + | - | - | - | - | + | + | [^60^](#_ENREF_60) |
| K55 | - | - | + | + | - | - | - | - | - | + | + | - | - | + | - | + | + | [^61^](#_ENREF_61) |
| K56 | - | - | + | + | - | - | - | - | - | - | - | + | + | + | + | + | - | [^62^](#_ENREF_62) |
| K57 | + | + | - | - | - | - | - | - | - | - | + | - | - | + | + | - | - | [^18^](#_ENREF_18) |
| K58 | - | + | - | - | + | + | + | - | - | + | + | + | + | + | - | + | + | [^63^](#_ENREF_63) |
| K59 | + | + | - | - | - | - | - | - | - | + | - | - | - | + | - | + | + | [^64^](#_ENREF_64) |
| K60 | + | + | - | - | - | - | - | - | - | - | - | - | - | + | - | + | + | [^65^](#_ENREF_65) |
| K61 | + | + | - | - | - | - | - | - | - | - | + | - | - | + | - | + | + | [^7^](#_ENREF_7) |
| K62 | + | + | - | - | - | - | - | - | - | - | + | - | - | + | + | + | - | [^66^](#_ENREF_66) |
| K63 | - | + | - | - | + | + | + | - | - | - | + | - | - | + | + | - | - | [^67^](#_ENREF_67) |
| K64 | + | + | + | + | - | - | - | - | - | - | - | + | + | - | - | + | + | [^2^](#_ENREF_2) |
| K65 | N/A | + | N/A | +^b^ | N/A | - | - | N/A | - | N/A | - | N/A | + | N/A | - | N/A | + |  |
| K66 | + | + | - | - | - | - | - | - | - | - | - | - | + | + | + | + | - | [^68^](#_ENREF_68) |
| K67 | + | + | + | + | - | - | - | - | - | - | - | - | - | + | - | + | + | [^69^](#_ENREF_69) |
| K68 | + | + | - | - | - | - | - | - | - | - | - | + | + | + | + | - | - | [^70^](#_ENREF_70) |
| K69 | + | + | - | - | - | - | - | - | - | - | + | + | + | + | - | + | + | [^71^](#_ENREF_71) |
| K70 | - | - | + | + | - | - | - | - | - | - | - | + | + | + | + | + | - | [^72^](#_ENREF_72) |
| K71 | - | - | + | + | - | - | - | - | - | - | - | - | - | - | - | + | + | [^73^](#_ENREF_73) |
| K72 | - | - | + | + | - | - | - | - | - | - | - | + | + | - | - | + | + | [^74^](#_ENREF_74) |
| K74 | + | + | - | - | - | - | - | - | - | - | - | + | + | + | + | - | - | [^75^](#_ENREF_75) |
| K79 | - | - | + | + | - | - | - | - | - | - | - | - | - | + | + | + | - | [^76^](#_ENREF_76) |
| K80 | + | + | + | + | - | - | - | - | - | - | - | + | + | + | + | - | - | [^77^](#_ENREF_77) |
| K81 | - | - | + | + | - | - | - | - | - | - | - | - | - | + | + | - | - | [^78^](#_ENREF_78) |
| K82 | - | - | - | - | - | - | - | - | - | + | + | - | - | + | - | + | + | [^23^](#_ENREF_23) |
| KN1 | N/A | - | N/A | + | N/A | - | - | N/A | - | N/A | - | N/A | + | N/A | + | N/A | - |  |
| KN2 | N/A | - | N/A | - | N/A | - | - | N/A | - | N/A | - | N/A | - | N/A | + | N/A | - |  |

**Supplementary Table S4. Comparison of *cps* genes and predicted functions**

|  | **common genes in K1 and K58** | **common structures in K1 and K58** |
| --- | --- | --- |
|  | wcaI | β-D-GlcpA(1→4)α-L-Fucp |
|  | wclY | 2, 3-Pyr-β-D-GlcpA |
|  | **unique genes in K1 and K58** | **unique structures in K1 and K58** |
| K1 | wcsS | α-L-Fucp(1→3)β-D-Glcp |
| K58 | wcqS, wcqT | α-L-Fucp(1→3)α-D-Glcp  α-D-Galp(1→3)α-L-Fucp |
|  | **common genes in K2 and K13** | **common structures in K2 and K13** |
|  | wcuD, wclX, wcuF | β-D-Glcp(1→4)β-D-Manp  β-D-Manp(1→4)α-D-Glcp  β-D-Galp(1→4)α-D-GlcpA |
|  | **unique genes in K2 and K13** | **unique structures in K2 and K13** |
| K13 | wcoW | β-D-Galp(1→4)α-D-GlcpA |
| K13 | wcuL | 3, 4-Pyr-β-D-Galp |
|  | **common genes in K12 and K41** | **common structures in K12 and K41** |
|  | wcaA, wclN, wclM, wckF | β-D-Galf(1→6)α-D-Glcp  α-D-Glcp(1→3)α-L-Rhap  α-L-Rhap(1→3)α-D-Galp  β-D-GlcpA(1→3)β-D-Galf |
|  | **unique genes in K12 and K41** | **unique structures in K12 and K41** |
| K12 | wckG | β-D-Galf(1→4)β-D-GlcpA |
| K12 | wckH | 5, 6-Pyr-β-D-Galf |
| K41 | wcpT, wcpU | β-D-Glcp(1→6)α-D-Glcp((1→4)β-D-GlcpA |
|  | **common genes in K30 and K69** | **common structures in K30 and K69** |
|  | wcuD, wckP, wckO, wckN | β-D-Manp(1→4)β-D-Manp  β-D-Manp(1→4)β-D-Glcp  α-D-GlcpA(1→3)β-D-Manp  β-D-Galp(1→6)β-D-Manp |
|  | **unique genes in K30 and K69** | **unique structures in K30 and K69** |
| K30 | wcuL (73% identity) | 3, 4-Pyr-β-D-Galp |
| K69 |  | 4, 6-Pyr-β-D-Galp |
|  | **common genes in K74 and K80** | **common structures in K74 and K80** |
|  | wbaZ | α-D-Manp(1→3)β-D-Galp |
|  | wcuD, wcuE | α-D-Manp(1→2)α-D-Manp  α-D-GlcAp(1→3)α-D-Manp |
|  | **unique genes in K74 and K80** | **unique structures in K74 and K80** |
| K74 | wcoW | β-D-Galp(1→4)α-D-GlcAp |
| K74 | wcuL | 4, 6-Pyr-β-D-Galp |
| K80 | wcuS | β-L-Rhap(1→4)α-D-GlcAp |
| K80 | wcuN | 3, 4-Pyr-β-L-Rhap |
|  | **common genes in K57 and K68** | **common structures in K57 and K68** |
|  | wbaZ | α-D-Manp(1→3)β-D-Galp |
|  | wckX, wckW | α-D-GalpA(1→2)α-D-Manp  α-D-Manp-(1→4)α-D-GalpA |
|  | **unique genes in K57 and K68** | **unique structures in K57 and K68** |
| K68 | wclV | 4, 6-Pyr-α-D-Manp |

**Supplementary Table S5**. Primers used in this study

| Primer name | Sequence | Position | Purpose or reference |
| --- | --- | --- | --- |
| pre-galF-F | GAGCCGCTGAATAACCTGAA | upstream of *galF* | *cps* 5’PCR[^16^](#_ENREF_16) |
| yegH | ggcgcgacgtcataatactg | *yegH* | *cps* 5’PCR[^16^](#_ENREF_16) |
| wzi(+1) R | TTGTACAAGATCCATTTTCAGC | *wzi* | *cps* 5’PCR |
| KP-wzc-CR1 | TTCAGCTGGATTTGGTGG | *wzc* | *cps* 5’PCR[^79^](#_ENREF_79) |
| KP-wzc-CR2 | GCTTCCATCATTGCAAAATG | *wzc* | *cps* 5’PCR |
| KP-wza-CF2 | GGGTTTTTATCGGGTTGTAC | *wza* | *cps* PCR |
| CPS-1 | GCTGGTAGCTGTTAAGCCAGGGGCGGT AGCG | upstream of *wzi* | *cps* PCR[^80^](#_ENREF_80) |
| rCPS | TATTCATCAGAAGCAGCACGCAGCTGG GAGAAGCC | *gnd* | *cps* PCR[^80^](#_ENREF_80) |
| rCPS2 | GCGCTCTGGCTGGTCCATTTACCGGTC CCTTTG | *gnd* | *cps* PCR[^80^](#_ENREF_80) |
| gnd 1R | CATCGGTGATCTTTTGCAGGAACTGCGCACGAATG | *gnd* | *cps* PCR |
| gnd 1F | GTTGTCGGTATGGCTGTGATGG | *gnd* | *cps* PCR |
| gnd+580F | ATGCAGCTGATTGCTGAAGC | *gnd* | *cps* PCR |
| post gnd R | GATGACCATCGGTTCATGGA | *manC* | *cps* 3’PCR[^16^](#_ENREF_16) |
| ugd | cgcgttcgggttgatctttg | *ugd* | *cps* 3’PCR[^16^](#_ENREF_16) |

References:

1. Dutton, G.G., Parolis, H. & Parolis, L.A. A structural investigation of the capsular polysaccharide of Klebsiella K14. *Carbohydr Res* **140**, 263-75 (1985).

2. Merrifield, E.H. & Stephen, A.M. Structural studies on the capsular polysaccharide from Klebsiella serotype K64. *Carbohydr Res* **74**, 241-57 (1979).

3. Ørskov, I. & Ørskov, F. Serotyping of Klebsiella. *Methods Microbiol.* **14**, 143-64 (1984).

4. Pieroni, P., Rennie, R.P., Ziola, B. & Deneer, H.G. The use of bacteriophages to differentiate serologically cross-reactive isolates of Klebsiella pneumoniae. *J Med Microbiol* **41**, 423-9 (1994).

5. Riser, E., Noone, P. & Poulton, T.A. A new serotyping method for Klebsiella species: development of the technique. *J Clin Pathol* **29**, 296-304 (1976).

6. Dutton, G.G., Ng, S.K., Parolis, L.A., Parolis, H. & Chakraborty, A.K. A re-investigation of the structure of the capsular polysaccharide of Klebsiella K10. *Carbohydr Res* **193**, 147-55 (1989).

7. Rao, A.S. & Roy, N. Structural studies on Klebsiella type 61 capsular polysaccharide. *Carbohydr Res* **67**, 449-56 (1978).

8. Lindberg, B., Lindh, F., Lonngren, J. & Nimmich, W. Structural studies of the capsular polysaccharide of Klebsiella type 33. *Carbohydr Res* **70**, 135-44 (1979).

9. Dutton, G.G. & Lim, A.V. Structural investigation of the capsular polysaccharide of Klebsiella serotype K35. *Carbohydr Res* **145**, 67-80 (1985).

10. Murcia, A. & Rubin, S.J. Reproducibility of an indirect immunofluorescent-antibody technique for capsular serotyping of Klebsiella pneumoniae. *J Clin Microbiol* **9**, 208-13 (1979).

11. Chuang, Y.P., Fang, C.T., Lai, S.Y., Chang, S.C. & Wang, J.T. Genetic determinants of capsular serotype K1 of Klebsiella pneumoniae causing primary pyogenic liver abscess. *Journal of Infectious Diseases* **193**, 645-54 (2006).

12. Fung, C.P. *et al.* A 5-year study of the seroepidemiology of Klebsiella pneumoniae: high prevalence of capsular serotype K1 in Taiwan and implication for vaccine efficacy. *The Journal of infectious diseases* **181**, 2075-9 (2000).

13. Shu, H.Y. *et al.* Genetic diversity of capsular polysaccharide biosynthesis in Klebsiella pneumoniae clinical isolates. *Microbiology* **155**, 4170-83 (2009).

14. Fevre, C. *et al.* PCR-based identification of Klebsiella pneumoniae subsp. rhinoscleromatis, the agent of rhinoscleroma. *PLoS Negl Trop Dis* **5**, e1052 (2011).

15. Fang, C.T. *et al.* Klebsiella pneumoniae genotype K1: an emerging pathogen that causes septic ocular or central nervous system complications from pyogenic liver abscess. *Clinical infectious diseases : an official publication of the Infectious Diseases Society of America* **45**, 284-93 (2007).

16. Pan, Y.J. *et al.* Capsular polysaccharide synthesis regions in Klebsiella pneumoniae serotype K57 and a new capsular serotype. *Journal of Clinical Microbiology* **46**, 2231-40 (2008).

17. Ho, J.Y. *et al.* Functions of some capsular polysaccharide biosynthetic genes in Klebsiella pneumoniae NTUH K-2044. *PLoS One* **6**, e21664 (2011).

18. Geyer, H., Himmelspach, K., Kwiatkowski, B., Schlecht, S. & Stirm, S. Degradation of bacterial surface carbohydrates by virus-associated enzymes. *Pure & Appl. Chem.* **55**, 637-53 (1983).

19. Dutton, G.G., Parolis, H., Joseleau, J.P. & Marais, M.F. The use of bacteriophage depolymerization in the structural investigation of the capsular polysaccharide from Klebsiella serotype K3. *Carbohydr Res* **149**, 411-23 (1986).

20. Knirel, Y.A. *et al.* Structure of the capsular polysaccharide of Klebsiella ozaenae serotype K4 containing 3-deoxy-D-glycero-D-galacto-nonulosonic acid. *Carbohydr Res* **188**, 145-55 (1989).

21. van Dam, J.E. *et al.* A bacteriophage-associated lyase acting on Klebsiella serotype K5 capsular polysaccharide. *Carbohydr Res* **142**, 338-43 (1985).

22. Dutton, G.G., Stephen, A.M. & Churms, S.C. Structural investigation of Klebsiella serotype K7 polysaccharide. *Carbohydr Res* **38**, 225-37 (1974).

23. Jansson, P.E. *et al.* Structural studies of the capsular polysaccharides from Klebsiella types 8 and 82, a reinvestigation. *Carbohydr Res* **175**, 103-9 (1988).

24. Isaac, D.H., Atkins, E.D.T. & Stirm, S. Molecular structure for microbial polysaccharides: conformation of the Klebsiella serotype K9 capsular polysaccharide. *Int. J. Biol. Macromol.* **3**, 165-170 (1981).

25. Thurow, H., Choy, Y.M., Frank, N., Niemann, H. & Stirm, S. The structure of Klebsiella serotype II capsular polysaccharide. *Carbohydr Res* **41**, 241-55 (1975).

26. Beurret, M., Joseleau, J.P., Vignon, M., Dutton, G.G. & Savage, A.V. Proof of the occurrence of 5,6-O-(1-carboxyethylidene)-D-galactofuranose units in the capsular polysaccharide of Klebsiella K12. *Carbohydr Res* **189**, 247-60 (1989).

27. Parolis, H., Parolis, L.A. & Whittaker, D.V. Re-investigation of the structure of the capsular polysaccharide of Klebsiella K15 using bacteriophage degradation and inverse-detected NMR experiments. *Carbohydr Res* **231**, 93-103 (1992).

28. Chakraborty, A.K., Friebolin, H., Niemann, H. & Stirm, S. Primary structure of the Klebsiella serotype 16 capsular polysaccharide. *Carbohydr Res* **59**, 525-530 (1977).

29. Dutton, G.G. & Folkman, T.E. Structural investigation of the capsular polysaccharide of Klebsiella serotype K17. *Carbohydr Res* **80**, 147-61 (1980).

30. Dutton, G.G.S., Mackie, K.L. & Yang, M.T. Structural Investigation of Klebsiella Serotype-K18 Polysaccharide. *Carbohydrate Research* **65**, 251-263 (1978).

31. Beurret, M., Vignon, M. & Joseleau, J.P. Structural investigation of the capsular polysaccharide from Klebsiella K19 by chemical and N.M.R. analyses. *Carbohydr Res* **157**, 13-25 (1986).

32. Choy, Y.M. & Dutton, G.G.S. Structure of Capsular Polysaccharide of Klebsiella K-Type 20. *Canadian Journal of Chemistry-Revue Canadienne De Chimie* **51**, 3015-3020 (1973).

33. Allen, P.M., Williams, J.M., Hart, C.A. & Saunders, J.R. Identification of two chemical types of K21 capsular polysaccharide from klebsiellae. *J Gen Microbiol* **133**, 1365-70 (1987).

34. Dutton, G.G.S., Mackie, K.L., Savage, A.V. & Stephenson, M.D. Structural Investigation of Capsular Polysaccharide of Klebsiella Serotype K23. *Carbohydrate Research* **66**, 125-131 (1978).

35. Choy, Y.M., Dutton, G.G.S. & Zanlungo, A.M. Structure of Capsular Polysaccharide of Klebsiella K-Type 24. *Canadian Journal of Chemistry-Revue Canadienne De Chimie* **51**, 1819-1825 (1973).

36. Niemann, H., Kwiatkowski, B., Westphal, U. & Stirm, S. Klebsiella serotype 25 capsular polysaccharide: primary structure and depolymerization by a bacteriophage-borne glycanase. *J Bacteriol* **130**, 366-74 (1977).

37. Difabio, J. & Dutton, G.G.S. Structural Investigation of the Capsular Polysaccharide of Klebsiella Serotype-K26. *Carbohydrate Research* **92**, 287-298 (1981).

38. Churms, S.C., Merrifield, E.H. & Stephen, A.M. The molecular structure of the capsular polysaccharide from Klebsiella type 27. *Carbohydr Res* **81**, 49-58 (1980).

39. Curvall, M., Lindberg, B. & Lonngren, J. Structural studies of the capsular polysaccharide of Klebsiella type 28. *Carbohydr Res* **42**, 95-105 (1975).

40. Lindberg, B., Lindh, F., Lonngren, J. & Sutherland, I.W. Structural studies of the capsular polysaccharide of Klebsiella type 30. *Carbohydr Res* **76**, 281-4 (1979).

41. Cheng, C.C., Wong, S.L. & Choy, Y.M. The structure of the capsular polysaccharide of Klebsiella K-type 31. *Carbohydr Res* **73**, 169-74 (1979).

42. Bebault, G.M., Dutton, G.G.S., Funnell, N.A. & Mackie, K.L. Structural Investigation of Klebsiella Serotype-K32 Polysaccharide. *Carbohydrate Research* **63**, 183-192 (1978).

43. Joseleau, J.P., Michon, F. & Vignon, M. Structural investigation of the capsular polysaccharide from Klebsiella serotype K-34 and its characterization by N.M.R. spectroscopy. *Carbohydr Res* **101**, 175-85 (1982).

44. Dutton, G.G. & Mackie, K.L. Structural investigation of Klebsiella serotype K36 polysaccharide. *Carbohydr Res* **55**, 49-63 (1977).

45. Jansson, P.E., Lindberg, B., Manca, M.C., Nimmich, W. & Widmalm, G. Structural studies of the capsular polysaccharide from Klebsiella type 38: a reinvestigation. *Carbohydr Res* **261**, 111-8 (1994).

46. Anderson, A.N., Parolis, H., Dutton, G.G. & Leek, D.M. Klebsiella serotype K39: structure of an unusual capsular antigen deduced by use of a viral endoglucosidase. *Carbohydr Res* **167**, 279-90 (1987).

47. Nath, R.K. & Chakraborty, A.K. Structural studies on the capsular polysaccharide of Klebsiella serotype K40. *Eur J Biochem* **162**, 439-43 (1987).

48. Beurret, M., Joseleau, J.P., Dutton, G.G. & Savage, A.V. Homologous and heterologous reactions of bacteriophages phi 41 and phi 12 on the capsular polysaccharides from Klebsiella K41 and K12. *Carbohydr Res* **189**, 237-46 (1989).

49. Aereboe, M., Parolis, H. & Parolis, L.A. Klebsiella K43 capsular polysaccharide: primary structure and depolymerisation by a viral-borne endoglycanase. *Carbohydr Res* **248**, 213-23 (1993).

50. Dutton, G.G. & Folkman, T.E. Structural investigation of the capsular polysaccharide of Klebsiella serotype K44. *Carbohydr Res* **78**, 305-15 (1980).

51. Dutton, G.G.S., Difabio, J.L. & Zanlungo, A.B. Structure of the Capsular Polysaccharide of Klebsiella Serotype-K45. *Carbohydrate Research* **106**, 93-100 (1982).

52. Okutani, K. & Dutton, G.G. Structural investigation of Klebsiella serotype K46 polysaccharide. *Carbohydr Res* **86**, 259-71 (1980).

53. Bjorndal, H., Lindberg, B., Lonngren, J., Rosell, K.G. & Nimmich, W. Structural studies of the Klebsiella type 47 capsular polysaccharide. *Carbohydr Res* **27**, 373-8 (1973).

54. Joseleau, J.P. & Marais, M.F. The structural repeating-unit of the capsular polysaccharide from Klebsiella serotype K48. *Carbohydr Res* **179**, 321-6 (1988).

55. Joseleau, J.P. Structural investigation of the capsular polysaccharide of Klebsiella serotype K 49. *Carbohydr Res* **142**, 85-92 (1985).

56. Altman, E. & Dutton, G.G. Structure of the capsular polysaccharide of Klebsiella serotype K50. *Carbohydrate Research* **118**, 183-194 (1983).

57. Chakraborty, A.K., Dabrowski, U., Geyer, H., Geyer, R. & Stirm, S. Primary structure of the Klebsiella serotype-51 capsular polysaccharide. *Carbohydr Res* **103**, 101-5 (1982).

58. Bjorndal, H., Lindberg, B., Lonngren, J., Meszaros, M. & Thompson, J.L. Structural studies of the capsular polysaccharide of Klebsiella type 52. *Carbohydr Res* **31**, 93-100 (1973).

59. Dutton, G.G. & Paulin, M. Structure of the capsular polysaccharide of Klebsiella serotype K53. *Carbohydr Res* **87**, 107-17 (1980).

60. Dutton, G.G.S. & Merrifield, E.H. The Capsular Polysaccharide from Klebsiella Serotype K54 - Location of the O-Acyl Groups, and a Revised Structure. *Carbohydrate Research* **105**, 189-203 (1982).

61. Bebault, G.M. & Dutton, G.G.S. Structural Investigation of Capsular Polysaccharide of Klebsiella Serotype-55. *Carbohydrate Research* **64**, 199-213 (1978).

62. Choy, Y.M. & Dutton, G.G.S. Structure of Capsular Polysaccharide of Klebsiella K-Type 56. *Canadian Journal of Chemistry-Revue Canadienne De Chimie* **51**, 3021-3026 (1973).

63. Dutton, G.S. & Savage, A.V. Structural investigation of the capsular polysaccharide of klebsiella serotype K58. *Carbohydr Res* **84**, 297-305 (1980).

64. Lindberg, B., LonngrenJ & Ruden, U. Structural studies of the capsular polysaccharide of Klebsiella type 59. *Carbohydr Res* **42**, 83-93 (1975).

65. Dutton, G.G. & Di Fabio, J. The capsular polysaccharide of Klebsiella serotype K60; a novel, structural pattern. *Carbohydr Res* **87**, 129-39 (1980).

66. Dutton, G.G. & Yang, M.T. Structural investigation of Klebsiella serotype K62 polysaccharide. *Carbohydr Res* **59**, 179-92 (1977).

67. Joseleau, J.P. & Marais, M.F. Structure of the capsular polysaccharide of Klebsiella K-type 63. *Carbohydr Res* **77**, 183-90 (1979).

68. Jansson, P.E., Lindberg, B., Lonngren, J., Ortega, C. & Nimmich, W. Structural Studies of the Capsular Polysaccharide of Klebsiella Type-66. *Carbohydrate Research* **132**, 297-305 (1984).

69. Dutton, G.G.S. & Karunaratne, D.N. Structural Investigation of the Capsular Polysaccharide of Klebsiella Serotype-K67. *Carbohydrate Research* **119**, 157-169 (1983).

70. Dutton, G.G., Parolis, H. & Parolis, L.A. The structural elucidation of the capsular polysaccharide of Klebsiella K68. *Carbohydr Res* **152**, 249-59 (1986).

71. Hackland, P.L., Parolis, H. & Parolis, L.A. A structural investigation of the capsular polysaccharide of Klebsiella K69. *Carbohydr Res* **172**, 209-16 (1988).

72. Dutton, G.G. & Mackie, K.L. Structural investigation of Klebsiella serotype K70 polysaccharide. *Carbohydr Res* **62**, 321-35 (1978).

73. Jackson, G.E., Ravenscroft, N. & Stephen, A.M. The use of bacteriophage-mediated depolymerisation in investigations of the structure of the capsular polysaccharide from Klebsiella serotype K71. *Carbohydr Res* **200**, 409-28 (1990).

74. Choy, Y.M. & Dutton, G.G.S. Structure of Capsular Polysaccharide of Klebsiella-K-Type 72 - Occurrence of 3,4-O-(1-Carboxyethylidene)-L-Rhamnose. *Canadian Journal of Chemistry-Revue Canadienne De Chimie* **52**, 684-687 (1974).

75. Dutton, G.S. & Paulin, M. Structure of the capsular polysaccharide of Klebsiella serotype K74. *Carbohydr Res* **87**, 119-27 (1980).

76. Dutton, G.G. & Lim, A.V. Structure of the capsular polysaccharide of Klebsiella serotype K79. *Carbohydr Res* **144**, 263-76 (1985).

77. Dutton, G.S. & Karunaratne, D. Structural investigation of the capsular polysaccharide of Klebsiella serotype K80. *Carbohydr Res* **134**, 103-14 (1984).

78. Curvall, M., Lindberg, B. & Lonngren. Structural studies of the capsular polysaccharide of Klebsiella type 81. *Carbohydr Res* **42**, 73-82 (1975).

79. Pan, Y.J. *et al.* Capsular types of Klebsiella pneumoniae revisited by wzc sequencing. *PLoS One* **8**, e80670 (2013).

80. Brisse, S., Issenhuth-Jeanjean, S. & Grimont, P.A. Molecular serotyping of Klebsiella species isolates by restriction of the amplified capsular antigen gene cluster. *J Clin Microbiol* **42**, 3388-98 (2004).
